# Phenazine Methosulfate Rewires Mitochondrial Redox Circuits to Restore Membrane Potential and ATP Synthesis Under ETC Blockade in Glioblastoma Cells

**DOI:** 10.3390/antiox15060749

**Published:** 2026-06-13

**Authors:** Andrius Kleinauskas, Marianna Canonaco, Tine Therese Henriksen Raabe, Elin Ryan, Petras Juzenas, Beata Grallert, Aspasia Valiraki, Athanasios Papakyriakou, Theodossis A. Theodossiou

**Affiliations:** 1 Department of Physics, University of Oslo, P.O. Box 1048 Blindern, N-0316 Oslo, Norway; andriukl@uio.no (A.K.); marican@fys.uio.no (M.C.); 2 Department of Radiation Biology, Institute for Cancer Research, Oslo University Hospital, Montebello, N-0379 Oslo, Norway; tinraa@ous-hf.no (T.T.H.R.); somezz@ous-hf.no (E.R.); petrasj@ous-hf.no (P.J.); beata@ous-hf.no (B.G.); 3 Institute of Biosciences & Applications, National Centre for Scientific Research “Demokritos”, Ag. Paraskevi, 15341 Athens, Greece; aspasiavaliraki8@gmail.com (A.V.); thpap@bio.demokritos.gr (A.P.)

**Keywords:** phenazine methosulfate, mitochondrial redox, cellular bioenergetics, electron transport chain, mitochondrial dysfunction, electron transfer

## Abstract

Mitochondrial electron transport chain (ETC) dysfunction is a major driver of bioenergetic failure, redox imbalance, and drug toxicity, yet strategies to restore oxidative phosphorylation under ETC blockade remain limited. Redox-active small molecules could, in principle, shuttle electrons from NADH to distal ETC components and oxygen, thereby modulating both respiration and reactive oxygen species (ROS) formation. Here, we show that the enzyme-independent redox cycler phenazine methosulfate (PMS) rewires mitochondrial redox circuits and restores respiration in human glioblastoma cells and cell-free systems under ETC inhibition. At subtoxic concentrations, PMS acutely increased oxygen consumption and mitochondrial superoxide generation via NADH–PMS–O_2_ redox cycling, while restoring mitochondrial membrane potential and ATP synthesis under ETC blockade, and shifting metabolism away from glycolytic lactate production. This profile is consistent with a protective redox-bypass role, distinct from the pro-apoptotic effects reported following high-dose, prolonged PMS exposure. The PMS-driven restoration of electron flow, mitochondrial membrane potential, and respiratory ATP synthesis under inhibition of Complex I (rotenone), III (antimycin A and myxothiazol), and/or IV (cyanide) is consistent with direct cytochrome *c* reduction, as demonstrated herein, and engagement of multiple ETC redox centers, including coenzyme Q_10_. In metformin-treated cells, PMS reversed suppression of respiration and lactate accumulation, outperforming existing redox-bypass strategies. These findings identify PMS-driven redox cycling as a previously unrecognized chemical redox-bypass mechanism that both regenerates mitochondrial bioenergetics and reshapes ROS production, suggesting a potential approach to counteract drug- and toxin-induced mitochondrial dysfunction and to exploit redox vulnerabilities in cancer.

## 1. Introduction

Mitochondrial oxidative phosphorylation (OXPHOS) is a central determinant of cellular bioenergetics, redox homeostasis, and survival, and its disruption is increasingly recognized as both a hallmark and a therapeutic target in human disease [1,2]. In cancer, chronic mitochondrial dysfunction and electron leakage from the respiratory chain contribute to sustained reactive oxygen species (ROS) production, while in toxicology, a range of xenobiotics and drugs directly impair electron transport chain (ETC) complexes, acutely compromising ATP synthesis and organ function [3,4].

In tumors, mitochondrial abnormalities and an imbalanced antioxidant network generate elevated ROS that can promote proliferation, angiogenesis, and therapy resistance, but also render malignant cells selectively vulnerable to further oxidative insults [3,5]. Cancer cells frequently rely on aerobic glycolysis (Warburg phenotype) while maintaining a leaky or partially impaired ETC, such that additional oxidative pressure can exhaust their antioxidant capacity and trigger cell death [6]. This dual role of ROS has led to the notion of a “double-edged sword” in cancer biology, where altering ROS levels can either promote tumor progression or be exploited therapeutically through a tumoricidal action [5].

Several clinically relevant agents exemplify how ETC inhibition underlies both therapeutic effects and dose-limiting toxicity. Metformin, the most widely used drug for type II diabetes, inhibits ETC complex I, leading to increased glycolysis, lactate accumulation, and a risk of lactic acidosis in susceptible patients [7,8]. Likewise, amiodarone, an effective antiarrhythmic, inhibits complexes I and II, and markedly reduces ATP production, contributing to cardiotoxic and hepatotoxic side effects [9,10]. In acute cyanide poisoning, direct inhibition of complex IV prevents oxygen reduction to water, abruptly collapsing mitochondrial respiration with life-threatening consequences; current antidotes are limited by slow onset, interference with oxygen transport, or the need for specialized administration [11]. These examples highlight an unmet need for strategies that can transiently bypass or “jump-start” blocked ETC segments to preserve OXPHOS and avoid adverse reactions.

One emerging concept is the use of redox-active small molecules as artificial electron shuttles that bridge NAD(P)H pools and distal ETC components, thereby restoring electron flow and ATP production despite complex-specific blockade [12]. Quinonoid compounds such as menadione/ascorbate (Apatone) and methylene blue (MB) have been explored as redox mediators, yet they typically depend on specific enzymatic systems (e.g., DT-diaphorase) or show limited efficacy under certain inhibitory contexts, such as metformin-induced complex I inhibition or amiodarone toxicity [13,14]. Succinate prodrugs like NV118 can partially restore respiration by feeding complex II, but their capacity to re-establish ATP synthesis and normalize glycolysis is constrained by the remaining integrity of downstream ETC complexes [15].

Phenazine derivatives offer an attractive alternative as enzyme-independent redox cyclers capable of shuttling electrons between cellular redox couples and molecular oxygen. In prokaryotes, endogenous phenazines can function as bona fide electron carriers; for instance, methanophenazine in Methanosarcina mazei functions as a membrane-embedded electron carrier, linking dehydrogenases to downstream cofactors and driving proton translocation for ATP synthesis [16,17,18]. In biochemical systems, the artificial phenazine phenazine methosulfate (PMS) has been extensively used to couple NADH or NADPH oxidation to reduction of oxygen as well as acceptor dyes such as tetrazolium salts, often in the absence of dedicated enzymes [19,20]. Enzyme-free NADH/O_2_ systems with PMS have been quantitatively characterized [21,22], in contrast to menadione-type quinones, which typically depend on enzymatic reduction by DT-diaphorase and subsequent autoxidation [13,14]. This strictly redox-substrate–driven, rather than enzyme-dependent, process suggests that PMS could serve both as a potent ROS generator and as an electron shuttle in cells with sufficient NAD(P)H pools.

Hua et al. reported that prolonged exposure of malignant melanoma cells to 10 μM PMS induced apoptotic cell death, mitochondrial damage, loss of mitochondrial membrane potential (ΔΨ_m_), impaired respiration, and superoxide generation [20]. The acute bioenergetic consequences of PMS exposure in intact tumor cells remain, however, incompletely defined. This knowledge gap is particularly relevant for glioblastoma multiforme (GBM), an aggressive brain tumor with notoriously abnormal metabolism and high oxidative stress [23]. GBM cells often harbor compromised antioxidant systems and defective mitochondria, which could render them especially vulnerable to exogenous redox-cycling agents that amplify ROS generation [24,25]. At the same time, the ability of such agents to bypass conventional ETC bottlenecks and sustain ΔΨ_m_ and OXPHOS raises the possibility of using them as tools to manipulate mitochondrial function or as adjuncts in strategies targeting mitochondrial vulnerabilities. Moreover, the widespread use of PMS as an electron mediator in dehydrogenase-coupled assays underscores the need to probe its potential implication in mitochondrial electron transport and cellular respiration.

In this study, we characterized the acute effects of PMS on mitochondrial bioenergetics and redox regulation in GBM cells and cell-free systems. Subtoxic concentrations of PMS significantly altered mitochondrial respiration and glycolytic activity across multiple GBM cell lines, revealing a distinct impact on cellular energy metabolism. In isolated, cell-free systems, PMS drove rapid, cell-independent NADH oxidation coupled to oxygen consumption, consistent with redox cycling between PMS and molecular oxygen, a process that generated superoxide anions. In cellular contexts, PMS-mediated electron transfer partially maintained mitochondrial membrane potential under inhibition of the electron transport chain, indicating a bypass mechanism for sustaining mitochondrial polarization. PMS exposure further supported mitochondrial ATP production under inhibited glycolysis, implying a compensatory role in energy maintenance. Finally, PMS/NADH enabled the direct rapid reduction of cytochrome *c*, indicating the possibility for electron delivery to downstream respiratory redox centers. Taken together, phenazine methosulfate (PMS) thus exhibits a bimodal profile in cells: at prolonged exposure and higher concentrations, PMS can act predominantly as a pro-oxidant, potentially leading to cancer cell death via excessive superoxide formation at higher concentrations (>10 μM). In contrast, under acute, subtoxic conditions and in the presence of sufficient reducing equivalents, PMS (≤10 μM) can function as an electron shuttle that restores electron flow through distal ETC components, maintains membrane potential, and supports respiratory ATP synthesis despite upstream complex blockade, with potential therapeutic value in acute mitochondrial impairment. In this work, we focus on the latter acute, subtoxic “redox bypass” activity of PMS, while recognizing that at higher, sustained doses, PMS may eventually promote ROS-mediated cytotoxicity, especially to cancer cells.

## 2. Materials and Methods

### 2.1. Chemicals and Reagents

Cytochrome *c* (cyt *c*), phenazine methosulfate (PMS), β-nicotinamide adenine dinucleotide hydrate (NADH), sodium pyruvate, oligomycin A (OLIGO), rotenone (ROT), carbonyl cyanide 4-(trifluoromethoxy)phenylhydrazone (FCCP), antimycin A (ANTIA), myxothiazol (MYXO), potassium cyanide (KCN), thiazolyl blue tetrazolium bromide (MTT), dimethyl sulfoxide (DMSO), superoxide dismutase from bovine erythrocytes (SOD), tetramethylrhodamine methyl ester perchlorate (TMRM), metformin, nitroblue tetrazolium chloride (NBT), 2-Deoxy-D-glucose (2DG), D-glucose, Dulbecco’s Phosphate Buffered Saline with MgCl_2_ and CaCl_2_ (PBS^+^), Trizma hydrochloride, and Trizma base were purchased from Merck (KGaA, Darmstadt, Germany). D-Luciferin Firefly potassium salt was purchased from Xenogen (now part of PerkinElmer, Shelton, CT, USA). Fetal bovine serum (FBS) was purchased from Biowest (Nuaillé, France). RPMI Medium 1640 without Phenol Red, L-glutamine, penicillin, streptomycin, trypsin, and MitoSOX™ Red were purchased from Thermo Fisher Scientific (Waltham, MA, USA). Seahorse XF Media and Calibrant were purchased from Agilent (Santa Clara, CA, USA).

### 2.2. Cell Culture

All parental cell lines (human GBM T98G (ATCC-CRL-1690), LN18 (ATCC-CRL-2610), M059K (ATCC-CRL-2365), U87 (ATCC-HTB-14), and human prostate C4-2) were originally obtained from ATCC. All cell lines including the luciferase-transfected ones (vide infra) were cultured in phenol red-free RPMI 1640 supplemented with 10% fetal bovine serum (FBS), 100 U/mL penicillin, 100 μg/mL streptomycin, and 2 mM L-glutamine at 37 °C in a humidified atmosphere of 5% CO_2_.

### 2.3. Construction of Cell Lines Expressing Luciferase

The PSA promoter region containing the Androgen Response Elements (AREs) and an upstream enhancer [construct PSA-64-s [26]] was synthetized (GeneArt, ThermoFisher Technologies, Waltham, MA, USA) and inserted into the pGL4.10 (Promega #E6651) plasmid upstream of firefly luciferase as a Kpn1-HindIII fragment. The PSA.luciferase fragment was subsequently subcloned into a pENTR vector as a Kpn1-Xba1 fragment.

To generate a lentiviral vector not containing a promoter, the lentiviral vector pLenti CMV Puro DEST (w118-1), a gift from Eric Campeau & Paul Kaufman [Addgene plasmid # 17452; http://n2t.net/addgene:17452 (accessed on 25 May 2026); [27]] was cut with Cla1 and PshA1, filled in, then circularized to create a promoter-less pLenti-puro-DEST.

Finally, PSA-64-s.luciferase was cloned into the pLenti-puro-DEST by Gateway cloning. The resulting plasmid was introduced into C4-2 cells by lentiviral transduction. The C4-2 cell line constitutively expresses the androgen receptor (AR) even without androgen treatment, and thus the expression of luciferase driven by the AR-dependent PSA promoter is constitutive. The resulting cell line was designated C4-2^PSA.LUC^.

RRL.sin.cPPT.SFFV/Flag-Firefly luciferase.IRES-puro.WPRE (MT05) was a gift from Caroline Goujon [Addgene plasmid # 139446 [28]] and was transduced into T98G cells, designated T98G^SFFV.LUC^.

### 2.4. Cytotoxicity Measurements

LN18, U87, M059K, and T98G cells (1.5 × 10^4^ per well) were seeded in 96-well plates and allowed to attach overnight in a humidified environment at 37 °C, 5% CO_2_. Cells were then incubated for 2 h with increasing concentrations of PMS (0–50 μM) in complete medium. Twenty-four hours after PMS removal, cell viability was assessed using standard MTT assays. Briefly, culture medium was replaced with 100 μL complete medium containing 1 mg/mL MTT, and cells were incubated for 1.5–2 h. The MTT solution was then removed and 100 μL DMSO was added to dissolve formazan crystals, followed by shaking for 10 min. Absorbance was measured at 561 nm using a Tecan Spark plate reader (Tecan Group Ltd., Männedorf, Switzerland). Blank wells (medium plus DMSO, no cells) were subtracted from all readings. Each condition was tested in at least six parallel wells.

### 2.5. Seahorse Experiments

Seahorse experiments with cells. Mitochondrial respiration in the form of oxygen consumption rate (OCR) and glycolysis in the form of extracellular acidification rate (ECAR) were measured using an XFe96 extracellular flux analyzer (Santa Clara, CA, USA). Cells were seeded at 3 × 10^4^ cells per well in Seahorse 96-well plates in 100 μL complete RPMI 1640 and incubated overnight at a 37 °C, 5% CO_2_ humidified atmosphere. The last row of each plate contained medium only (no cells) as background controls. One hour before measurement, complete medium was replaced with unbuffered XF RPMI (pH 7.4) supplemented with 10 mM glucose, 2 mM L-glutamine, and 2 mM sodium pyruvate, and plates were incubated at 37 °C in a non-CO_2_ incubator.

Inhibitors and test compounds were injected through the cartridge ports at the following final concentrations: OLIGO and FCCP, 1.5 or 2 μM; ANTIA/ROT (±MYXO), 2 or 4 μM; KCN, 1.2 mM. PMS was applied at 2, 5, or 10 μM. Metformin was dissolved directly in Seahorse assay medium and injected in real time during the run at a final concentration of 5 mM.

Seahorse experiments with solutions. For cell-free assays, XF RPMI medium (pH 7.4, without supplements) was pre-equilibrated at 37 °C for 1 h and then used to prepare NADH solutions at final concentrations of 10 or 50 mM. Either medium alone or NADH-containing medium was added to Seahorse 96-well plates. PMS was injected into medium-only or NADH containing wells to final concentrations of 5 or 10 μM; control wells received medium instead of PMS.

### 2.6. Flow Cytometry Assays

For all flow cytometry measurements, assay medium consisted of PBS^+^, supplemented with 1% D-glucose and 1 mM sodium pyruvate. Cells were grown in T175 flasks as described in the cell culture section. Before analysis, cells were trypsinized, counted, and resuspended in PBS containing CaCl_2_ and MgCl_2_, 1 mM sodium pyruvate, and 1% D-glucose, at a final concentration of 2.6 × 10^6^ cells/mL. Aliquots of 100 µL (2.6 × 10^5^ cells) were transferred to flow cytometry tubes. All samples were kept at 37 °C in a water bath and protected from light.

Mitochondrial membrane potential was assessed using TMRM, and mitochondrial superoxide was detected with MitoSOX™ Red (Thermo Fisher Scientific, Waltham, MA, USA). Flow cytometry was performed on a CytoFLEX S instrument (Beckman Coulter Life Sciences, Indianapolis, IN, USA) using CytExpert software (version 2.1). Data were analyzed with FlowJo software (version 10, Treestar, Ashland, OR, USA). Live cells were gated based on forward scatter area (FSC-A) and side scatter area (SSC-A), and single viable cells were further selected using FSC-A versus FSC-W. TMRM or MitoSOX™ Red fluorescence was recorded for the gated population under all experimental conditions, and the geometric mean ± standard error was used for data presentation. For experiments with repeated measurements, the mean ± standard error of the mean (SEM) was reported, $SEM=\sigma /\sqrt{n}$, where *n* denotes the number of independent measurements.

### 2.7. Analysis of Mitochondrial Membrane Potential by TMRM Flow Cytometry

The following experimental conditions were analyzed:

(i) Unstained control cells (background; negative control for TMRM);
(ii) TMRM-stained cells, incubated with TMRM (20 nM, 15 min);
(iii) Drug-treated cells, incubated with mitochondrial inhibitors (MYXO, ANTIA, ROT; 2 µM each, 5 min), followed by TMRM staining (20 nM, 15 min);
(iv) PMS-treated cells, incubated with PMS (5 or 10 µM, 5 min), followed by TMRM staining (20 nM, 15 min);
(v) Drug + PMS-treated cells, incubated with mitochondrial inhibitors (2 µM each, 5 min) prior to PMS addition (5 or 10 µM, 5 min), followed by TMRM staining (20 nM, 15 min);
(vi) FCCP-treated cells, incubated with FCCP (1 µM, 5 min), followed by TMRM staining (20 nM, 15 min).

TMRM fluorescence was detected in the propidium iodide (PI) channel (561 nm laser excitation, 585/42 nm bandpass emission filter). A total of 20,000 live, single cells were analyzed per sample.

### 2.8. Analysis of Mitochondrial Superoxide by MitoSOX™ Red Flow Cytometry

The following experimental conditions were analyzed:

(i) Unstained control cells (background; negative control for MitoSOX™ Red);
(ii) MitoSOX-stained cells, incubated with MitoSOX™ Red (2.5 µM, 20 min);
(iii) PMS-treated cells, incubated with PMS (5 or 10 µM, 10 min), followed by MitoSOX™ Red staining (2.5 µM, 20 min).

MitoSOX™ Red fluorescence was recorded in the Violet610 channel (405 nm excitation, 610/20 nm bandpass emission filter). For each condition, 20,000 live single cells were evaluated.

### 2.9. Determination of Respiratory ATP in Luciferase-Transduced T98G and C4-2 Cells

Luciferase-expressing T98G^SFFV.LUC^ and C4-2^PSA.LUC^ cells were incubated in T175 flasks at least overnight before experiments. Prior to measurements, the cells were detached by trypsinization, counted, and resuspended in assay medium at 1 × 10^6^ cells/mL. Aliquots of 100 µL were transferred into assay tubes and maintained at 37 °C in a humidified incubator with 5% CO_2_. Assay medium consisted of PBS^+^, supplemented with 1% FBS and 1 mM sodium pyruvate.

Luciferin was added as the substrate for and initiator of the luciferase–ATP chemiluminescence reaction in the presence of oxygen, and the resulting signal was used as a readout of cellular ATP levels. To inhibit glycolysis, 2DG was added prior to measurements at a final concentration of 50 mM (2 h for T98G^SFFV.LUC^ and 1 h for C4-2^PSA.LUC^), in darkness to prevent PMS degradation and measurement artifacts [29]. This ensured that the measured signal primarily reflected respiratory ATP. Mitochondrial ETC inhibitors MYXO, ANTIA, and ROT (7–10 μM) were used to block complexes I (ROT) and III (ANTIA, MYXO). NADH (20 or 33 mM) was added to support redox cycling with PMS (8–10 μM).

Luminescence was recorded using a portable luminometer (ABELmeter™, Knight Scientific Ltd., Plymouth, UK) equipped with a photomultiplier tube (PMT) and dedicated acquisition software. The final reaction volume varied depending on sequential additions. Four experimental conditions were used:

1. Luciferin (300 μM) + mitochondrial inhibitors (10 μM each);
2. Luciferin (300 μM) + mitochondrial inhibitors (8 μM each) + NADH (20 mM);
3. Luciferin (300 μM) + mitochondrial inhibitors (8 μM each) + NADH (20 mM) + PMS (10 μM);
4. Luciferin (200 μM) + mitochondrial inhibitors (7 μM each) + NADH (33 mM) × 2 + PMS (8 μM).

Raw luminescence signals were corrected for instrumental artifacts using custom Python scripts (version 3.12.2, available in the Supplementary Materials). Signals were segmented to account for acquisition-related discontinuities, normalized to their respective maximum values, and reassembled to preserve signal continuity and the temporal profile of the biological response. Raw luminescence traces are provided in Supplementary Figure S1.

### 2.10. Real-Time Registration of Cytochrome c Reduction by PMS/NADH

A 2 mM stock solution of cyt *c* was prepared in Tris buffer (50 mM, pH 8.5). This buffer was also used for the NBT±SOD-PMS/NADH experiments (vide infra). The reaction mixture was assembled in a glass cuvette by combining NADH (1.8 mM) and cyt *c* (300 µM). The cuvette was placed on a magnetic stirrer and continuously mixed with a stir bar. The reaction was initiated by adding PMS to a final concentration of 5 μM.

The reaction was recorded in time-lapse mode at an effective frame rate of 30 frames/s. Video data were analyzed using custom Python scripts (Supplementary Materials). Reaction time was estimated from the complete color change of the solution. The a* channel in CIELab color space was used, as this space separates chromatic information (a*, b*) from luminance (L*), making the analysis more robust to illumination drift. The signal was modeled in two segments: a linear phase (reaction) and a post-reaction plateau. The reaction end point was determined by minimizing the sum of the errors coming from the fit of both segments. Absorbance spectra of the solutions (cyt *c* and NADH) before and after PMS addition (endpoint) were recorded with a Shimadzu UV1900i spectrophotometer (Shimadzu, Stockholm, Sweden) to verify the redox state of cyt *c*.

### 2.11. NBT Reduction by PMS/NADH in the Presence and Absence of SOD

NBT reduction was monitored spectrophotometrically in Tris buffer (50 mM, pH 8.5) with or without Cu,Zn-SOD (2000 U) in a final volume of 1 mL. NBT (80 µM) was added first, followed by NADH (320 µM) and finally PMS (5 µM). The solution was gently mixed by pipetting after each addition before recording. Absorbance at 560 nm was measured using the Shimadzu UV1900i UV–Vis spectrophotometer (Shimadzu, Stockholm, Sweden) with an accumulation time of 0.2 s, a total acquisition time of 5 min, and a data interval of 2 s.

### 2.12. Statistical Analysis

Unless otherwise specified, errors are reported as 1 SEM. Flow cytometry data are presented as geometric mean ± SE; for experiments with multiple repetitions, geometric means from each repeat were averaged and the SE of the mean is shown. Statistical significance was evaluated using one-tailed Student’s *t*-tests and is denoted as ns (*p* > 0.05), * (*p* < 0.05), ** (*p* < 0.01), and *** (*p* < 0.001). All experiments were repeated at least 3 times.

### 2.13. Molecular Modeling

The models presented herein were generated based on the cryo-EM structures of mammalian complex I in the active state with Q_10_/NADH bound (PDB ID: 7V2C and 7VCE, resolved at 2.8–2.9 Å) [30] and the high-resolution X-ray structure of yeast complex III with bound cytochrome *c* in reduced state (PDB ID: 3CX5, resolved at 1.9 Å) [31]. The complexes were inserted into 1-palmitoyl-2-oleoyl-sn-glycero-3-phosphocholine (POPC) lipid bilayers generated using CHARMM-GUI server [32] without any modifications. The modeled NADH, PMS, and Q_10_ molecules were based on crystallographic structures and were manually placed into visually intuitive locations of the models, except for the cyt *c*-interacting PMS for which the docked pose was obtained using Autodock VINA [33].

## 3. Results and Discussion

### 3.1. Seahorse Metabolic Analyses with the in Situ Application of PMS in GBM Cell Lines

**Figure 1:**
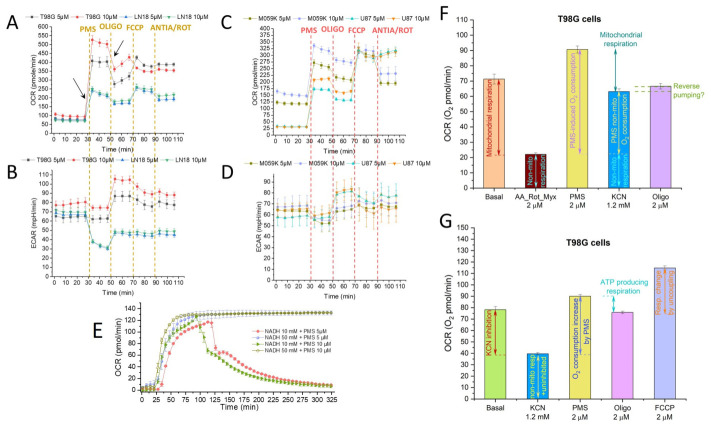
**Seahorse analysis of PMS redox cycling in cells and cell-free systems.** (**A**). Effect of PMS (5 and 10 μM) on the OCR of T98G and LN18 cells, followed by a mitochondrial stress test (OLIGO 1.5 μM, FCCP 1.5 μM, ANTIA/ROT 2 μM). (**B**). Effect of PMS (5 and 10 μM) on the ECAR of T98G and LN18 cells, followed by a mitochondrial stress test. (**C**). Effect of PMS (5 and 10 μM) on the OCR of M059K and U87 cells, followed by a mitochondrial stress test. (**D**). Effect of PMS (5 and 10 μM) on the ECAR of M059K and U87 cells, followed by a mitochondrial stress test. (**E**). OCR traces recorded after addition of PMS (5 and 10 μM) to cell-free solutions containing 10 or 50 mM NADH. (**F**). OCR in T98G cells under the following conditions: basal (light orange), after addition of complex I/III inhibitors (ANTIA, ROT, MYXO; 2 μM each, brown), after subsequent addition of PMS (2 μM, yellow), following addition of the complex IV inhibitor KCN (1.2 mM, blue), and after OLIGO (2 μM, magenta). (**G**). OCR in T98G cells under the following conditions: basal (light green), after addition of the complex IV inhibitor KCN (1.2 mM, blue), after subsequent addition of PMS (2 μM, yellow), after OLIGO (2 μM, pink), and after FCCP (2 μM, lilac). All error bars represent ±1 SEM. The experiments shown in Figure 1 were performed between 3 and 5 times (*n* = 3–5).

To assess the acute effects of PMS on mitochondrial bioenergetics, we administered PMS in real time to GBM cells during Seahorse analyses, followed by a standard Mito Stress Test (OLIGO, FCCP, and ROT plus ANTIA; Figure 1A–D). Subtoxic PMS concentrations were defined in preliminary toxicity assays (Figure S2), where GBM cell lines were exposed to PMS for 2 h (matching the exposure during Seahorse runs), and MTT viability was assessed 24 h after PMS removal. Up to 5 μM PMS was non-toxic in T98G, M059K, LN18, and U87 cells, while 10 μM PMS was also largely tolerated in T98G, M059K, and U87 cells. Based on these data, we employed PMS concentrations of 2, 5, and/or 10 μM throughout the study. Sensitivity to PMS differed between cell lines: LN18 cells were the most sensitive to 10 μM PMS, with 33 ± 2% cytotoxicity, whereas no statistically significant cytotoxicity was observed at 10 μM in T98G (7 ± 19%), M059K (20 ± 24%), or U87 (9 ± 19%) cells.

We additionally performed a long-term toxicity and cell tolerance experiment with PMS. GBM cells (T98G, U87, and M059K) were incubated with PMS (1, 2, and 5 μM) and cell viability was assessed at 24–144 h (1–6 days). The results are shown in Figure S3. T98G cells tolerated PMS well up to and including 5 μM, with no marked toxicity within experimental error. U87 cells tolerated up to 2 μM PMS with viabilities ≥ 75%; however, 5 μM caused incremental toxicity over time, reaching approximately 10% survival at day 3 (72 h), with partial recovery in the subsequent three days, rising to approximately 65% viability by day six. M059K cells were the most sensitive of the three lines to prolonged PMS exposure, showing substantial loss of viability even at 2 μM (approximately 35% viability at all timepoints), while viability at 5 μM was below 10% at all timepoints. Notably, even at 1 μM, viabilities were in the range of 75–85% across all assay timepoints. These results are in good agreement with our assertion that PMS cytotoxicity is highly cell-type dependent. T98G cells, which exhibit a higher metabolic profile—including elevated respiration more characteristic of normal cells—likely possess stronger antioxidant defenses and are therefore better able to withstand the ROS burden imposed by PMS. These findings further confirm the capacity of PMS to selectively kill Warburg-type cancer cells without conferring a fatal outcome on surrounding normal cells.

In Figure 1A, we show the effect of PMS on the OCR of T98G and LN18 cells. In T98G cells, 5 μM PMS increased basal OCR approximately 4-fold, and 10 μM PMS increased OCR by ~5-fold. In LN18 cells, both 5 and 10 μM PMS increased OCR by ~2.5-fold. Subsequent oligomycin addition decreased T98G OCR from ~400 to ~280 pmol/min (5 μM PMS) and from ~500 to ~360 pmol/min (10 μM PMS), i.e., a ~1.4–1.5-fold drop, reflecting ATP-linked respiration. In LN18 cells, oligomycin reduced OCR by ~1.3-fold for both PMS concentrations. FCCP addition after oligomycin increased T98G OCR by ~1.3-fold at 5 μM PMS, whereas at 10 μM PMS, no further increase (and even a slight decrease) was observed. This likely reflects an interplay between (i) the maximal respiratory capacity of the cells under PMS-induced OCR and (ii) the interaction between oxygen superoxide anions and the proton flux produced by the protonophore (vide infra). In LN18 cells, FCCP increased OCR by ~1.4-fold at both PMS concentrations, indicating that FCCP induced OXPHOS uncoupling and that the ETC retained additional capacity to maximize its electron flow and consequently OCR beyond that attained by PMS. After addition of ROT plus ANTIA, which inhibit the quinone-reducing sites of complexes I and III, blocking the overall electron transport, the OCR remained high (at the same level as after FCCP) in both T98G and LN18 cells, demonstrating that PMS sustained oxygen consumption independently of the ETC electron flow.

Figure 1B shows the ECAR corresponding to the OCR traces of Figure 1A. PMS addition reduced ECAR—more markedly in LN18 (~50%) and modestly in T98G (~5%)—consistent with a shift away from glycolysis as PMS-enhanced respiration supported a bigger part of the cellular ATP demand. This corroborates the conclusion that the PMS-induced OCR increases reflect genuine enhancement of mitochondrial respiration via elevated electron flux. The pronounced PMS-induced ECAR decline in LN18 cells may underlie their lower PMS tolerance, which might relate to their p53 deficiency [34], combined with a forced shift toward a more respiratory phenotype [35]. When OLIGO was applied, inhibition of respiratory ATP production triggered compensatory glycolysis, with ECAR increasing by ~35% in LN18 and ~40% in T98G cells.

Figure 1C,D show analogous experiments in M059K and U87 GBM cells. PMS induced a sharp OCR rise, by ~2–2.5-fold in M059K and by ~7-fold (5 μM PMS) to ~8-fold (10 μM PMS) in U87 cells. OLIGO reduced OCR in both lines (−35 pmol/min in M059K at 5 and 10 μM PMS; −50 pmol/min in U87 at 10 μM PMS and −35 pmol/min at 5 μM PMS). FCCP then increased OCR by stimulating proton conductance across the inner membrane and driving the ETC to higher flux. In M059K cells, maximal FCCP-stimulated OCR was similar to the OCR observed after 10 μM PMS (~320 pmol/min), while after 5 μM PMS, the FCCP-induced maximal OCR was ~50 pmol/min higher. In U87 cells, FCCP increased OCR above PMS levels by ~150 pmol/min (5 μM PMS) and ~120 pmol/min (10 μM PMS). ROT plus ANTIA, which should reduce OCR to non-mitochondrial levels, decreased M059K OCR from ~300 to ~195 pmol/min (5 μM PMS) and from ~315 to ~230 pmol/min (10 μM PMS). In contrast, U87 OCR did not decrease after ROT plus ANTIA at either PMS concentration, indicating that residual OCR in U87 was largely non-respiratory and driven by direct PMS–oxygen interactions leading to oxygen intermediates (vide infra), thereby masking the ETC-related OCR drop.

Basal ECAR in M059K and U87 cells was similar (60–70 mpH/min; Figure 1D). PMS (5 and 10 μM) caused modest ECAR decreases (~5 mpH/min in U87 and ~12 mpH/min in M059K), consistent with a partial shift from glycolytic to respiratory ATP production. OLIGO subsequently increased ECAR by ~15 mpH/min (M059K) and ~24 mpH/min (U87), again reflecting compensatory glycolysis in response to F_1_F_0_-ATP synthase inhibition.

In addition to the above experiments, we carried out a Seahorse experiment incorporating incremental PMS injections into T98G cells, all in the same experimental run.

The results are shown in Figure S4A. From the traces in that figure, it is evident that up to 5 μM PMS, there is a concentration-dependent increase in OCR, which drops upon addition of OLIGO in all cases. At PMS concentrations up to 5 μM, subsequent addition of FCCP causes a marked increase in OCR; however, at 5 and 10 μM PMS, this increase is small and approaches zero. Shutdown of the ETC by ANTIA and ROT produces a substantial decrease from the FCCP-stimulated OCR at concentrations up to 2 μM PMS, but incurs little additional change at 5 and 10 μM PMS. These observations already indicate that at higher PMS concentrations the FCCP-stimulated OCR is lower, and the non-ETC OCR at 5 and 10 μM PMS is substantially lower than that at 2 μM PMS. Both effects are consistent with our theory of superoxide disproportionation to H_2_O_2_ and O_2_ following FCCP addition (see below), which may indeed operate at higher PMS concentrations.

**Figure 2:**
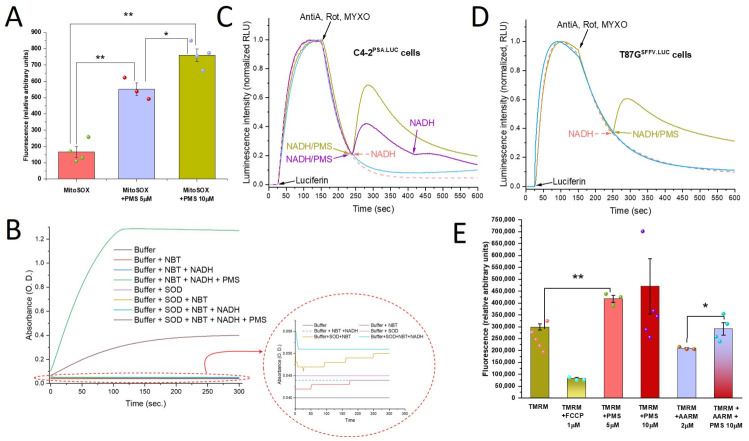
**PMS-induced superoxide production, respiratory ATP generation, and mitochondrial membrane potential.** (**A**). Flow cytometry analysis of mitochondrial superoxide in cells treated with PMS (5 or 10 μM, 10 min) and MitoSOX™ Red (2.5 μM, 20 min). MitoSOX fluorescence was excited at 405 nm and collected with a 610/20 nm bandpass filter. Data represent mean ± 1 SEM from ≥3 independent experiments. Statistical significance was assessed by one-tailed Student’s *t*-tests and is indicated as * (*p* < 0.05), ** (*p* < 0.01). (**B**). Absorbance kinetics of NBT reduction at 560 nm (accumulation time 0.2 s, total acquisition time 5 min, data interval 2 s). Background-level kinetics are shown magnified in the inset with dotted lines. (**C**). Bioluminescence profile of luciferase-transduced C4-2^PSA.LUC^ cells. Cells were pretreated with 2-DG (50 mM, 1 h) to block glycolytic ATP production. Traces shown are (1) luciferin (300 μM) plus mitochondrial ETC inhibitors MYXO/ANTIA (complex III) and ROT (complex I) (10 μM each), solid cyan line; (2) as in (1) with the addition of NADH (20 mM), dashed red line; (3) as in (1) with the addition of PMS/NADH (10 μM/20 mM), solid dark yellow line; (4) as in (3) with an extra NADH bolus (200 μM luciferin, 7 μM inhibitors, 8 μM PMS), solid magenta line. (**D**). Bioluminescence profile of luciferase-transduced T98G^SFFV.LUC^ cells. Cells were pretreated with 2-DG (50 mM, 2 h). Traces shown are (1) luciferin (300 μM) plus mitochondrial ETC inhibitors MYXO/ANTIA (complex III) and ROT (complex I) (10 μM each), solid cyan line; (2) as in (1) with the addition of NADH (20 mM), dashed red line; (3) as in (1) with the addition of PMS/NADH (10 μM/20 mM), solid dark yellow line. (**E**). Flow cytometry analysis of mitochondrial membrane potential in T98G cells treated with TMRM (20 nM, dark yellow column), FCCP (1 μM) + TMRM (gradient yellow column), ANTIA + ROT + MYXO (2 μM each) + TMRM (light blue column), PMS alone (5 μM, light red column; 10 μM, dark red column), and inhibitors + TMRM + PMS (gradient dark red/light blue column). Errors denote (a) mean ± 1 SEM from ≥2 independent measurements for TMRM alone (20 nM, 15 min), 5 μM PMS (5 min) + TMRM (20 nM, 15 min), 10 μM PMS (5 min) + TMRM (20 nM, 15 min), inhibitors (2 μM, 5 min) + TMRM (20 nM, 15 min), and inhibitors (2 μM, 5 min) + 10 μM PMS (5 min) + TMRM (20 nM, 15 min); (b) 1 SEM of the geometric mean for FCCP (1 μM, 5 min) + TMRM (20 nM, 15 min). Statistical significance was evaluated by one-tailed Student’s *t*-tests and is indicated as * (*p* < 0.05), ** (*p* < 0.01). The experiments shown in Figure 2 were repeated at least 3 times (*n* ≥ 3).

Using the data in Figure S4A, we further calculated the following derived parameters: PMS-BASAL OCR, representing the net increase in basal respiration attributable to PMS at each concentration; PMS-OLIGO, representing the drop in PMS-stimulated OCR upon addition of Oligomycin, thereby blocking respiratory ATP production and revealing what proportion of the PMS-enhanced OCR was coupled to ATP synthesis; and FCCP-OLIGO, indicating the net increase in OCR following FCCP addition at each PMS concentration. From these data, shown in Figure S4B, the net PMS-induced OCR (PMS-BASAL) increased up to 2 μM and subsequently reached a plateau at 5 and 10 μM PMS. The PMS-OLIGO values followed a very similar trend, indicating that an increasing amount of respiratory ATP was produced at increasing PMS concentrations—fully supporting our chemiluminescence data (see below, Figure 2C,D). Importantly, this outcome precludes any possibility of PMS-induced uncoupling, as the OXPHOS enhancement driven by PMS electron donation remained coupled to ATP production by the F_1_F_0_-ATP synthase.

The behavior of FCCP-OLIGO was strikingly different. While FCCP induced a marked increase in OCR at lower PMS concentrations (0.2–1 μM, with 2 μM representing the inflection point), it was able to induce only a small OCR increase at the higher concentrations of 5 and 10 μM PMS. This observation further supports our theory of spontaneous superoxide disproportionation at these concentrations (see below), which returns a substantial amount of oxygen to the medium and thereby effectively reduces the measured OCR.

As will be shown in the next section, the PMS-driven OCR was partly due to the reduction of O_2_ and formation of reactive oxygen species.

### 3.2. Seahorse Metabolic Analyses in NADH-Containing Solutions with in Situ PMS Addition

The cellular experiments suggested that PMS acted both on the mitochondrial ETC and directly on ambient oxygen via redox cycling, presumably with NADH as the electron donor. NADH is the high-energy electron carrier oxidized at complex I (NADH dehydrogenase) during OXPHOS, cycling between NADH and NAD^+^. Driven by the cellular Seahorse data, we next examined PMS-driven oxygen consumption in cell-free solutions containing NADH (10 or 50 mM). PMS was injected in real time at final concentrations of 5 or 10 μM, as in the cellular assays (Figure 1A–D), and the resulting OCR traces are shown in Figure 1E.

In 10 mM NADH, PMS injection produced a delayed but marked rise in OCR. With 10 μM PMS, OCR began to increase approximately 1 min after injection and reached a maximum of ~126 pmol/min at 93 min of total assay time. Beyond this time point, OCR declined in a biphasic manner, with an initial rapid phase (slope ~−3) up to ~112 min, followed by a slower decay from 112 to 324 min. With 5 μM PMS in 10 mM NADH, OCR began to rise ~5 min after injection and peaked at ~118 pmol/min at 114 min, again followed by a biphasic decline: a rapid drop to ~60 pmol/min by ~130 min, a modest shoulder around 135 min, and a slower decrease thereafter until 324 min.

In 50 mM NADH, both PMS concentrations induced a rapid rise in OCR to a plateau of ~130 pmol/min, which was sustained until the end of the assay (324 min). The main difference between 5 and 10 μM PMS was the time required to reach this plateau. At 5 μM PMS, OCR reached the plateau at ~124 min (~100 min after injection), whereas at 10 μM PMS, the rise was steeper and the plateau was attained by ~85 min (~61 min after injection). NADH alone did not alter OCR, which remained at medium background levels, and PMS injections in the absence of NADH also left OCR at background.

These observations indicate that oxygen consumption in solution required both NADH and PMS; i.e., molecular oxygen did not redox-cycle detectably with the NADH/NAD^+^ couple in the absence of PMS. We conclude that PMS is reduced by NADH, and the reduced PMS then transfers electrons to oxygen, generating superoxide $\left(O_{2}^{.-}\right)$, while both NADH and reduced PMS are concomitantly oxidized. Importantly, each 3 min OCR measurement was preceded by a 3 min mixing step, allowing re-equilibration of dissolved oxygen in the wells. Under these conditions, OCR displayed an apparent ceiling of ~130–135 pmol/min, which indicated a ceiling for the $\left(O_{2}^{.-}\right)$ production, too, and was independent of NADH concentration once a sufficient NADH pool was present to sustain PMS redox cycling.

The magnitude of PMS-induced OCR in solution was comparable to that observed in some GBM cell lines. Treating the 50 mM NADH solution as an effectively infinite NADH reservoir, analogous to the continuously regenerated NADH pool in cells, allows a simple comparison: arithmetically adding the maximal OCR in NADH solutions (~130 pmol/min; Figure 1E) to the basal OCR of LN18 cells (~75 pmol/min; Figure 1C) yields ~205 pmol/min, close to the OCR measured in LN18 cells after 10 μM PMS (~220 pmol/min). By contrast, in T98G cells, PMS increased OCR by up to ~450 pmol/min, far exceeding the ~130 pmol/min ceiling observed in PMS–NADH solutions. Within cells, several endogenous redox-active components can generate $O_{2}^{.-}$ during redox cycling apart from the direct interaction of PMS with oxygen. Coenzyme Q_10_ and other quinones, for example, can be reduced to semiquinone intermediates that donate electrons to oxygen, producing superoxide [36,37]. Depending on the availability of transition metals, $O_{2}^{.-}$ can be particularly damaging, via Haber–Weiss chemistry downstream its dismutation to hydrogen peroxide, ultimately yielding highly reactive hydroxyl radicals.

### 3.3. Detection of Mitochondrial Superoxide Using MitoSOX™ Red and Flow Cytometry

To further confirm $O_{2}^{.-}$ generation via PMS-driven intracellular reduction of molecular oxygen, we measured fluorescence intensity of the mitochondrial superoxide–sensitive probe MitoSOX™ Red by flow cytometry. T98G cells were pre-treated with PMS (5 or 10 μM) and subsequently loaded with MitoSOX™ Red; fluorescence intensities are shown in Figure 2A.

Under basal conditions, T98G cells exhibited low mitochondrial superoxide levels (~168 Relative Fluorescence Units, RFU). Pre-treatment with PMS for 5 min before MitoSOX™ Red loading caused a marked, concentration-dependent increase in fluorescence. At 5 μM PMS, mitochondrial superoxide–associated signal increased to ~550 RFU (~3.3-fold), while 10 μM PMS elevated the signal to ~760 RFU (~4.5-fold). These findings, consistent with previous observations by Hua et al. [20], demonstrate a dose-dependent rise in mitochondrial $O_{2}^{.-}$ following PMS exposure.

Because MitoSOX™ Red selectively accumulates in mitochondria, these data indicate that PMS promotes intramitochondrial superoxide formation [38]. This enhanced mitochondrial $O_{2}^{.-}$ production underlies part of the PMS-enhanced OCR detected in cellular Seahorse assays (Figure 1A–D,F,G) and reflects the activity of the intracellular NADH/PMS redox system. In contrast, in the cell-free experiments shown in Figure 1E, exogenous NADH is required to sustain PMS–NADH/NAD^+^ redox cycling and drive oxygen reduction.

### 3.4. Study of the PMS–NADH System in the Presence of NBT with or Without SOD by Absorbance Kinetics

To further demonstrate that the PMS–NADH redox pair generates superoxide in the presence of oxygen, we used NBT as a terminal electron acceptor for $O_{2}^{.-}$, as in previous studies [39,40,41]. Upon reduction, NBT forms a purple formazan that can be monitored spectrophotometrically. In our experiments, TRIS buffer with or without Cu,Zn-SOD (2000 IU) was supplemented sequentially with NBT (80 μM), NADH (320 μM), and finally PMS (5 μM). The results are shown in Figure 2B. In the absence of SOD, baseline absorbance remained low in buffer alone, buffer + NBT, and buffer + NBT + NADH. The addition of PMS, however, caused a steep absorbance increase to ~1.3 OD within ~2 min.

In the presence of SOD, no increase in absorbance was observed after adding NBT and NADH, while PMS addition yielded only a modest rise to ~0.4 OD, reached more slowly (~4 min). This corresponds to ~70% inhibition of NBT reduction by SOD. Thus, there was no appreciable spontaneous NBT reduction in buffer, nor after NADH addition alone; PMS was required to initiate NBT reduction, indicating that NADH could not directly reduce NBT under these conditions. Ponti et al. [39] concluded that, in an NBT/PMS/NADH system, PMS/NADH rapidly reduces oxygen to superoxide as well as NBT directly, while later work by Picker and Fridovich [40] and by Van Noorden and Butcher [41] suggested that $O_{2}^{.-}$ production can be mediated via NBT in its anionic form.

In our experiments, the strong SOD sensitivity of NBT reduction highlights the central role of oxygen-derived $O_{2}^{.-}$ in NBT formazan formation. Because our primary aim was to confirm PMS/NADH–oxygen interaction, we did not further dissect potential direct PMS/NADH–NBT reactions. Nonetheless, our data imply that, at most, ~30% of NBT reduction could be SOD-insensitive (i.e., independent of superoxide), whereas the remaining majority must have proceeded via oxygen redox chemistry.

Additional findings support this interpretation: (i) PMS increased oxygen consumption in NADH-containing, cell-free Seahorse assays in an NADH dose–dependent manner (Figure 1E), in the absence of NBT, strongly indicating direct oxygen reduction by the PMS/NADH redox system; and (ii) PMS activated MitoSOX™ Red in intact cells in a PMS-dependent manner, evidently using intracellular NADH pools (Figure 2A). Together, these results not only link PMS to $O_{2}^{.-}$ formation, but also indicate that PMS reaches mitochondria and generates superoxide there. This conclusion is strengthened by the short lifetime and limited diffusion radius of superoxide [42,43,44], which effectively precludes a major contribution from superoxide produced in distant cellular compartments.

### 3.5. Seahorse Metabolic Analyses of PMS Addition to GBM Cells After ETC Inhibition at Complexes I, III, and IV

Having established that PMS enhances cellular respiratory activity, we next asked whether PMS can functionally compensate for ETC inhibition at complexes I, III, and IV. We hypothesized that PMS might partially bypass blocked ETC segments and restore electron flow. To test this, we used T98G cells, in which PMS elicited the greatest increase in OCR, and acutely inhibited the ETC immediately after basal OCR measurements by adding ROT, ANTIA, and MYXO. This cocktail targeted complex III at both its quinone-reducing and quinol-oxidizing sites and complex I at its quinone-reducing site. We then added KCN to additionally inhibit complex IV, and finally OLIGO to assess whether the cells were still responsive. The results are shown in Figure 1F.

Following addition of ROT, ANTIA, and MYXO, the OCR decreased by ~70% relative to normalized basal respiration, with the remaining ~30% attributed to non-mitochondrial oxygen consumption. Subsequent addition of 2 μM PMS increased OCR by ~97%, raising it to ~27% above basal. As shown above, this PMS-induced increase reflected both an enhancement of electron flow and direct PMS-driven oxygen reduction to $O_{2}^{.-}$. The ensuing KCN addition reduced OCR by ~40%, likely representing the fraction of oxygen consumption still coupled to mitochondrial respiration. OLIGO then produced a modest ~5% OCR increase, which may reflect reverse operation of the F_1_F_0_-ATP synthase in an attempt to export protons from the mitochondrial matrix and support membrane potential.

In a separate experiment, we inhibited complex IV first by adding KCN immediately after recording basal OCR. This intervention reduced OCR by ~50%, leaving non-mitochondrial respiration and possibly some residual mitochondrial activity due to incomplete inhibition. Addition of 2 μM PMS then increased OCR by ~65% (to ~15% above basal), while subsequent OLIGO decreased OCR by ~20%, corresponding to the fraction respiration coupled to ATP synthesis. Finally, FCCP increased OCR by a further ~30% relative to the PMS condition, driving the ETC to its maximal respiratory rate.

Together, these experiments show that PMS can substantially compensate for impaired electron transport, even when complexes I and III or complex IV are inhibited. This raised two key questions for subsequent analyses: (1) Is the PMS-induced respiratory compensation linked to ATP production via OXPHOS? and (2) Does this compensation engage the mitochondrial proton pumps sufficiently to export H^+^ from the matrix and maintain or enhance mitochondrial membrane potential?

### 3.6. Bioluminescence Assays in C4-2 and T98G Cells Expressing Luciferase to Assess Intracellular ATP

To address the impact of PMS on ATP production, in addition to the evidence of PMS-induced respiratory ATP production in cells with uninhibited respiration shown in Figure S4, we performed bioluminescence assays in cells expressing luciferase, exploiting the strict dependence of the luciferin–luciferase reaction on intracellular ATP [45,46]. Because cellular ATP is primarily derived from OXPHOS and glycolysis, we preincubated cells with excess 2-DG to block glycolysis, thereby making the bioluminescent signal predominantly dependent on OXPHOS-derived ATP. The resulting data are shown in Figure 2C,D.

Figure 2C presents the results from C4-2^PSA.LUC^ cells. In all traces, the luminescence before luciferin addition was at background levels. Luciferin addition induced a rapid rise in bioluminescence to a plateau. Subsequent addition of complex I/III inhibitors (ROT, ANTIA, and MYXO) caused luminescence to decline progressively, reaching ~5% of the maximal value within ~130 s. This confirmed that the recorded signal reflected OXPHOS-generated ATP, which was depleted following ETC inhibition as expected. When a PMS/NADH mixture was then added, the luminescence intensity increased again, reaching ~60% of the initial plateau, indicating that the PMS/NADH redox pair provided a bypass electron flow capable of partially restoring ATP production via OXPHOS. In a separate experiment, a second NADH addition produced a further modest luminescence increase, despite the likely adverse impact of combined 2-DG and complex I and III inhibition on cellular homeostasis and viability.

A similar pattern was observed in T98G^SFFV.LUC^ cells (Figure 2D). After luciferin addition, luminescence reached a plateau, which dropped to ~10% of the maximal level upon addition of complex I/III inhibitors. Subsequent PMS/NADH addition increased luminescence by ~50%, again consistent with PMS-driven restoration of OXPHOS-dependent ATP synthesis.

In these experiments, exogenous NADH was required to drive PMS redox cycling, most likely because prolonged 2-DG exposure markedly reduced intracellular NADH:NAD^+^ ratios [47]. In addition, extracellular pyruvate in the medium likely acted as a redox sink, being reduced to lactate by lactate dehydrogenase in the cytosol and, consequently, rapidly oxidizing any remaining NADH to NAD^+^.

### 3.7. Study of Mitochondrial Membrane Potential Using TMRM and Flow Cytometry

To test whether PMS can drive the mitochondrial proton pumps that maintain the ΔΨ_m_ by exporting H^+^ from the matrix to the intermembrane space, we used TMRM staining and flow cytometry. TMRM accumulation in mitochondria is strongly dependent on ΔΨ_m_: when ΔΨ_m_ is low or dissipated and mitochondria are depolarized (e.g., by protonophores such as FCCP or by ETC inhibition), TMRM accumulation and fluorescence are low; when mitochondria are hyperpolarized, TMRM uptake and fluorescence increase accordingly. The results are shown in Figure 2E.

FCCP treatment reduced the basal TMRM signal by ~70%, and this depolarization was not reversed by PMS, whether applied as a pre-treatment or in co-incubation. In contrast, PMS alone markedly increased TMRM fluorescence (by ~40% with 5 μM PMS and ~60% with 10 μM PMS), indicating that PMS hyperpolarized mitochondria by enhancing proton pumping from the matrix into the intermembrane space. Inhibition of complexes I and III with ROT, ANTIA, and MYXO reduced TMRM signal by ~30%, consistent with a partial loss of ΔΨ_m_ due to impaired electron flow to the proton pumps of complexes I and III [48,49]. This decrease in ΔΨ_m_ was efficiently reversed by adding 10 μM PMS together with the inhibitors, restoring TMRM fluorescence to ~97% of basal levels.

These findings indicate that PMS can restore ΔΨ_m_ after ETC inhibition, but not after FCCP-induced uncoupling. In the former case, ΔΨ_m_ declines because a lack of electron flow limits proton extrusion by the ETC pumps; PMS can rectify this by supplying additional electron flux to the pumps, bypassing normal enzymatic pathways and restoring ΔΨ_m_. In the latter case, FCCP collapses ΔΨ_m_ by shuttling protons from the intermembrane space back into the matrix [50,51]. Under these conditions, even maximal activity of the proton pumps, reverse operation of F_1_F_0_-ATP synthase via ATP hydrolysis [52], and PMS-driven electron input are insufficient to overcome the continuous proton leak imposed by FCCP.

### 3.8. FCCP Effects on PMS-Stimulated OCR

Following the same logic, we can explain why FCCP further increases PMS-elevated OCR in some cell lines, has no effect in others, and even decreases OCR in a subset. When FCCP is added on top of PMS, it exerts two opposing effects: (i) it drives the ETC toward its maximal respiratory capacity, and (ii) as a protonophore, it floods the mitochondrial matrix with protons, which then react with PMS-generated superoxide in a two-step protonation/disproportionation sequence $O_{2}^{.-}+H^{+}⇄HO_{2}^{.}$ (1) and $2HO_{2}^{.}\to H_{2}O_{2}+\;O_{2}$ (2) [53,54]. The first effect (i) tends to increase OCR by pushing electron flux to its maximal level, whereas the second (ii) effectively returns ~50% of $O_{2}^{.-}$, back to molecular oxygen, thereby lowering net oxygen consumption.

In summary, FCCP superimposed on PMS can:

(i) Decrease OCR in cell lines where the ETC-driven increase is small relative to the PMS component, so that protonation/disproportionation of $O_{2}^{.-}$ dominates.
(ii) Increase OCR in cell lines where the maximal respiratory capacity dominates over superoxide protonation/disproportionation, particularly when the PMS-induced OCR elevation is modest.
(iii) Produce no apparent net change in OCR when these two effects are of similar magnitude and cancel each other, leaving the PMS-enhanced OCR unchanged.

A note on the above: Under coupled conditions, the mitochondrial matrix is maintained at pH ~7.8–8.0 by the ΔpH component of the protonmotive force [55,56], rendering essentially all matrix superoxide in its fully deprotonated O_2_•^−^ form (pKa of HO_2_•/O_2_•^−^ = 4.8 [57]) and making the first protonation step rate-limiting for non-enzymatic dismutation. Upon addition of FCCP, the protonophore abolishes the transmembrane ΔpH, equilibrating matrix pH toward the cytosolic value (~7.0–7.2) [56,58]. This proton influx increases the availability of H^+^ for the first protonation step (O_2_•^−^ + H^+^ → HO_2_•) [59], accelerating non-enzymatic dismutation alongside the constitutively active MnSOD pathway, and returning ~50% of consumed O_2_ to molecular form per dismutation cycle. In cell lines where the PMS-driven OCR component is large relative to maximal ETC capacity, this net return of O_2_ from superoxide dismutation is sufficient to decrease the observed total OCR despite FCCP-driven ETC acceleration.

### 3.9. Relation to Previous Work and Physiological Relevance

Our cellular Seahorse data are consistent with the classic findings of Ikehara et al. [60], and offer a mechanistic explanation for them. In that study, PMS stimulated cellular respiration and reduced lactate production, even in the presence of the inhibitors NaCN and ROT. Ikehara et al. also showed that cellular NADH was markedly oxidized by PMS or PMS plus NaCN/ROT, but not by the inhibitors alone, clearly indicating that PMS taps into the intracellular NADH pool, undergoes redox cycling, and donates electrons onward to the mitochondrial ETC.

Our metabolic analyses, including PMS-driven restoration of ΔΨ_m_ and respiratory ATP production after ETC inhibition, support the notion that PMS can at least partially substitute for, or supplement, a compromised, impaired, or inhibited ETC. There are natural precedents for phenazines functioning as electron carriers in place of quinones. For example, the archaeon *Methanosarcina mazei* Gö1 produces methanophenazine, a hydrophobic phenazine with a pentaisoprenoid side chain [16]. Methanophenazine is reduced by electrons derived from hydrogen or a reduced cofactor (an NADH analog in methanogens) via two dehydrogenases, one homologous to bacterial/mitochondrial NADH dehydrogenase. The reduced methanophenazine then donates electrons to a downstream cofactor [17], ultimately supporting proton translocation and generating a proton gradient used for ATP synthesis [18].

### 3.10. Direct PMS/NADH Reduction of Cytochrome c

**Figure 3:**
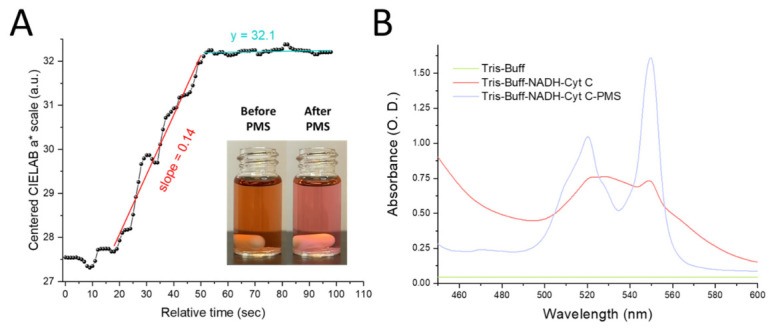
**Reduction of cytochrome *c* by the PMS/NADH redox system.** (**A**). Time-lapse analysis of the reaction between cytochrome *c* (300 μM) and PMS (5 μM)/NADH (1.8 mM) in 50 mM Tris buffer, pH 8.5. The signal was extracted from the a* channel of the CIELab color space. (**B**). Endpoint absorbance spectra of the cytochrome *c* + PMS + NADH mixture before (red trace) and after (lilac trace) PMS addition and reaction completion, compared with Tris buffer baseline (green). This experiment was performed at least 4 times (*n* ≥ 4).

Before investigating whether PMS action might be enzyme-mediated (e.g., acting as a substrate of complexes I or III, as previously explored for hypericin [61]), we examined direct PMS/NADH–cytochrome *c* interactions in solution. Cytochrome *c* (300 μM) was dissolved in 50 mM TRIS, pH 8.5, followed by addition of NADH (1.8 mM), and finally PMS (5 μM) under stirring. Upon PMS addition, the solution rapidly changed color (Figure 3, inset), consistent with cytochrome *c* reduction, as previously observed for PMS/NADH systems [40].

In the present work, we analyzed the kinetics in an unconventional manner by video recording the reaction and quantifying the time course of the CIELab a* channel (Figure 3A). Before PMS addition, we had a plateau region at ~27.5, followed by a sigmoidal increase that we fitted with a linear segment (slope ≈ 0.14), reaching a final plateau at ~32.1. The transition from lower to upper plateau corresponded to a reaction time of ~32 s. The reaction was essentially diffusion-limited: varying the stirring rate or reactant concentrations shortened the completion time to as little as ~3 s.

We then used spectrophotometry to confirm the redox state of cytochrome *c* before and after PMS addition (Figure 3B). After PMS, the 550 nm peak was strongly increased compared with the pre-PMS spectrum. The 550/540 nm absorbance ratios were ${\frac{550\;nm}{540\;nm}}_{before\;PMS}=1\;$ (low), while ${\frac{550\;nm}{540\;nm}}_{after\;PMS}=2.3\;$ (high), consistent with oxidized cytochrome *c* before, and reduced cytochrome *c* after [62]. These results indicate that PMS/NADH reduces cytochrome *c* directly and essentially instantaneously on contact. Given this rapid, non-enzymatic reduction, we did not pursue additional enzymatic assays, because the observed kinetics outpace typical enzyme-limited reactions.

**Figure 4:**
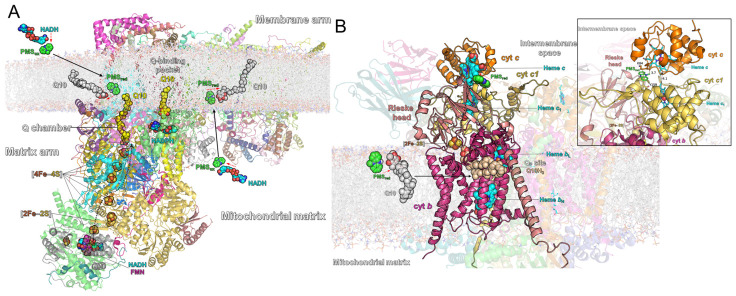
**Molecular models illustrating the PMS activity on the ETC components.** (**A**). Model of the NADH:ubiquinone oxidoreductase (complex I) highlighting the electron transport pathway (black dashed arrow) from the NADH/FMN pair bound at the NADH-oxidizing module through seven iron–sulfur clusters (2Fe–2S and 4Fe–4S) of the matrix arm to the Q chamber-bound ubiquinone (Q_10_, yellow C, red O atoms). The second bound Q_10_ modeled near a tightly bound NADPH molecule (cyan C, blue N, and red O atoms) is at a putative Q-binding pocket resolved in a recent cryo-EM structure [30]. PMS, modeled with green C atoms, can be reduced by free NADH at the intermembrane space, or at the mitochondrial matrix (red dashed arrow indicates the hydride transfer from the nicotinamide ring of NADH to the oxidized cationic PMS_ox_). The more lipophilic, reduced PMS (PMS_red_) can then transfer two electrons to membrane-accumulated Q_10_ molecules (white C atoms), bypassing the physiological electron transfer pathway of complex I. (**B**). Model of the mitochondrial cytochrome *bc*_1_ complex (complex III) with transiently bound soluble cytochrome *c* (cyt *c*) [31], highlighting the electron transfer pathway from ubiquinol (Q_10_H_2_, white C atoms) through the [2Fe–2S] cluster of the Rieske protein and cytochrome *c*1 (cyt *c*1) subunit to cyt *c*. PMS_red_ can facilitate either two-electron transfer to ubiquinone within the membrane, or direct electron transfer to cyt *c*. The solid arrow indicates passive diffusion of Q_10_ after reduction to Q_10_H_2_ into the Q_o_ site, similarly to the diffusion of PMS_red_ into the inner mitochondrial membrane shown in panel (**A**). Inset is a close-up view of the cyt *bc_1_*/cyt c interface with docked PMS, indicating two potential interacting residues (Lys84 of cyt *c* and Tyr159 of cyt *c*1) and the minimal distances of heme *c*/heme *c1* and PMS/heme *c* (Å).

The efficient reduction of cytochrome *c* by PMS/NADH provides a mechanistic framework for understanding the capacity of PMS to reduce additional redox centers within the mitochondrial ETC, including additional cytochromes, iron–sulfur clusters, and potentially coenzyme Q_10_ itself. Reduction of the oxidized, cationic PMS species (PMS_ox_, Figure 4) by NADH, whether in the intermembrane space or mitochondrial matrix, yields the lipophilic reduced form (PMS_red_, Figure 4), which can readily partition into the inner mitochondrial membrane. Within this environment, PMS_red_ is positioned to reduce membrane-embedded ubiquinone (Q_10_) to ubiquinol (Q_10_H2), effectively bypassing the physiological electron transfer pathway through NADH:ubiquinone oxidoreductase (complex I, Figure 4A). This process becomes particularly relevant when complex I activity is impaired, and oxidized Q_10_ accumulates in the membrane. Consequently, elevated levels of reduced Q_10_ can sustain electron flow to cytochrome *c* (cyt *c*) through the physiological activity of complex III (cytochrome *bc1*, Figure 4B). Docking analyses further support a direct interaction between reduced PMS and cyt *c*, providing docked poses in close proximity to its heme group (heme *c*, Figure 4B, inset), at distances consistent with efficient electron transfer and comparable to those observed for the heme group of cytochrome *c1*. Collectively, these observations indicate that PMS can sustain mitochondrial electron flow via two complementary routes: (i) direct reduction of Q_10_ within the inner mitochondrial membranes, or (ii) direct electron transfer to cyt *c* under conditions where complexes I or III are compromised. Potential interaction of PMS_red_ with other ETC components, including the iron–sulfur clusters ([2/4Fe–2/4S], Figure 4) and the Q_o_ site of complex III, cannot be ruled out, though these appear less physiologically significant compared with the direct reduction of Q_10_ or cyt *c*, especially during ETC inhibition.

Oxidative stress is widely considered both as a consequence and a driver of mitochondrial dysfunction. In tumors, dysfunctional mitochondria are a major source of oxidative stress due to elevated electron leak and inefficient coupling of electron and proton transfer to oxygen in the ETC, resulting in increased ROS generation [3,63]. Deficient antioxidant defenses (e.g., glutathione peroxidases, catalase, SOD) can render certain cell lines—particularly cancer cells—more vulnerable to ROS than others [64]. Numerous studies show that ROS-generating systems such as menadione/ascorbate exert stronger cytotoxic effects on glioblastoma cells than on normal cells [65,66,67], which are comparatively more tolerant of ROS exposure. A key advantage of PMS over menadione/ascorbate is that PMS does not require ascorbate coupling to generate ROS; it can directly use NADH both for ROS production and for electron transport. In the literature, PMS is predominantly characterized in enzyme-free NADH/O_2_ systems [21,22], whereas menadione-type quinones are mainly described in DT-diaphorase–driven reduction/autoxidation schemes [13,14], making direct comparisons of their ROS-producing properties indirect. The fact that PMS action is not strictly enzyme-dependent but rather linked to NAD(P)H pool size and redox state may represent a major functional advantage.

The most relevant report on PMS as an anticancer agent is by Hua et al. [20], where PMS redox reactions induced apoptotic death in malignant melanoma (A375) cells. PMS exposure upregulated heat shock, oxidative, and genotoxic stress response genes, and was associated with mitochondriotoxicity, impaired mitochondrial respiration, loss of ΔΨ_m_, and superoxide generation. Importantly, these adverse effects were observed after several hours of exposure to 10 μM PMS—a concentration that, in our hands, can produce slight cytotoxicity depending on the cell type. The mitochondrial respiratory impairment reported in that work, measured by Seahorse analysis, was likewise based on prolonged PMS preincubation. We suspect that PMS was not replenished during the Seahorse run, so the observed “respiratory toxicity” likely reflects emerging cell death in that particular line (A375) under extended PMS exposure rather than an immediate effect of PMS on intact bioenergetics.

Furthermore, Hua et al. showed that PMS-induced melanoma cytotoxicity was mediated by ROS. As discussed above, normal cells often tolerate substantially higher ROS loads than cancer cells. In cancer, ROS assume a dual role: moderate levels can promote progression by enhancing proliferation, migration, invasion, angiogenesis, and therapy resistance, whereas excessive ROS induce oxidative stress and cell death. ROS are therefore a classic double-edged sword in cancer biology, with their regulation critical to both tumor development and therapeutic response [68]. In this context, PMS can shift cancer cells from a Warburg-type, glycolysis-dominated metabolism toward a more respiratory state. Combined with their leaky ETC and already elevated ROS, this shift can drive depletion of antioxidant defenses and ultimately cell death [20]. Conversely, PMS can also rescue OXPHOS in normal cells with pharmacologically impaired or inhibited ETC.

One clinically relevant example involves complex I inhibition by metformin, the most widely used drug for type II diabetes [69]. A well-recognized drawback of metformin therapy is ETC complex I blockade, increased glycolysis, and consequent lactate accumulation leading to lactic acidosis [70]. In a study by Piel et al. [71], the redox agent methylene blue was used to bypass complex I and facilitate electron transfer from NAD(P)H to cytochrome *c* with the aim to alleviate lactic acidosis. While MB produced a dose-dependent increase in respiration in complex I–inhibited platelets (ROT), it failed to reverse metformin-induced ETC inhibition or reduce lactate production, in contrast to the cell-permeable succinate prodrug NV118, which was effective in that setting.

### 3.11. Seahorse Analyses of PMS in Metformin-Treated Cells: Lifting ETC Blockade and Reducing Lactate Production

**Figure 5:**
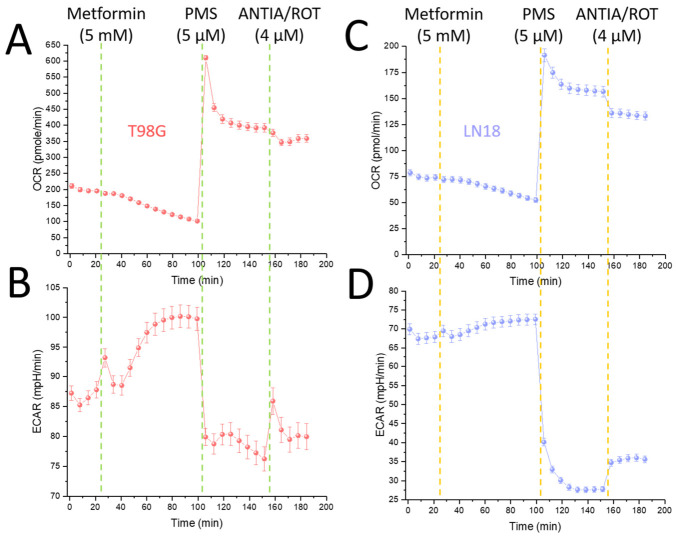
**Seahorse analysis of the effect of PMS on T98G and LN18 GBM cells under metformin inhibition.** (**A**). Oxygen consumption rate (OCR) of T98G cells under the following conditions: basal (untreated), after addition of metformin (5 mM), after subsequent addition of PMS (5 μM), and after ANTIA + ROT (4 μM). (**B**). Extracellular acidification rate (ECAR) of T98G cells corresponding to the OCR traces in panel (**A**). (**C**). OCR of LN18 cells under the following conditions: basal (untreated), after addition of metformin (5 mM), after subsequent addition of PMS (5 μM), and after ANTIA + ROT (4 μM). (**D**). ECAR of LN18 cells corresponding to the OCR traces in panel (**C**). Dashed lines denote the timepoint of each injection. All error bars represent ±1 SEM. This experiment was performed 3 times (*n* = 3).

Motivated by reports that agents such as NV118 can restore respiration under metformin-induced ETC inhibition, we examined whether PMS could similarly bypass metformin’s effects and attenuate the associated lactate production. We added metformin by injection during Seahorse runs to monitor the real-time decline in OCR, and then tested whether PMS could reverse this inhibition. The results are shown in Figure 5.

In T98G cells (Figure 5A), metformin addition caused a progressive decrease in OCR. Subsequent injection of 5 μM PMS caused OCR to surge from ~103 to ~611 pmol/min before stabilizing at ~393 pmol/min. Final addition of ANTIA plus ROT reduced OCR by ~45 pmol/min, reflecting the remaining mitochondrial respiration of T98G cells. This drop does not capture the PMS-driven component of OCR, which is insensitive to ETC inhibitors. Parallel experiments showed that metformin exerted an overall ~85% inhibitory effect on T98G OCR from its injection until the addition of ANTIA/ROT (~130 min).

The functional relevance of metformin inhibition and PMS rescue is supported by the corresponding ECAR changes reflecting glycolysis (Figure 5B). Metformin increased ECAR by ~12 mpH/min, indicating a compensatory rise in glycolysis in response to suppressed respiration and ATP loss. PMS addition produced a marked OCR increase that was accompanied by an ECAR decrease of ~20 mpH/min, consistent with PMS-enhanced respiratory ATP production that drove downregulation of glycolysis to avoid ATP overproduction. Subsequent ANTIA/ROT treatment increased ECAR by up to ~10 mpH/min (and ~5 mpH/min at steady state) as glycolysis again compensated for impaired mitochondrial ATP production. However, ECAR did not fully return to its pre-PMS level, suggesting that PMS still supported some OXPHOS-derived ATP after ETC inhibition.

A similar pattern was observed in LN18 cells (Figure 5C). Again, metformin exerted an inhibitory effect on the OCR in LN18 cells. PMS increased OCR by ~140 pmol/min to a peak, which then decayed to a steady state ~105 pmol/min above the metformin-depressed baseline—still substantial, but less dramatic than in T98G. ETC inhibition by ANTIA/ROT subsequently reduced OCR by ~24 pmol/min. From parallel experiments, metformin produced an overall ~75% inhibition of LN18 OCR from its initial injection up to ANTIA/ROT addition (~130 min).

In LN18 ECAR traces, metformin produced a modest ECAR increase (~5 mpH/min), whereas PMS addition induced a much larger ECAR decline (~45 mpH/min), greater than that observed in T98G. This suggests that PMS induced a more pronounced ATP surplus in LN18 cells, requiring a stronger glycolytic downshift to restore energy balance. After ANTIA/ROT ETC inhibition, ECAR increased by only ~8 mpH/min, indicating that PMS-induced enhancement of electron flow and respiratory ATP production remained dominant, limiting the need for further glycolytic compensation. Because ECAR reflects medium acidification driven largely by glycolytic lactate export, these findings indicate that PMS substantially reduced metformin-associated lactate production in both cell lines. On this basis, PMS appears more potent in alleviating metformin’s adverse effects on cellular respiration than NV118 [71], and thus merits further exploration for translational use.

Another potential application of PMS arises in the context of amiodarone therapy for cardiac arrhythmias [72]. While clinically valuable, amiodarone inhibits respiration at ETC complexes I and II and markedly decreases ATP generation [10,73]. In this setting, NV118 has been shown to partially restore respiration in platelets and HepG2 cells, but without documented ATP recovery [15]. In contrast, our data demonstrate that PMS not only augments and restores respiration under complex I, III, or IV inhibition, but also rescues respiratory ATP production (Figure 2C,D).

Especially under complex I inhibition, but also more broadly during respiratory chain blockade, the intracellular NADH/NAD^+^ ratio can rise to levels at which NADH itself becomes deleterious to cells [74]. In such situations, the NAD^+^ pool can be replenished by endogenous regenerative pathways, such as glycerol-3-phosphate biosynthesis [75]. A further advantage of using PMS under ETC blockade conditions is that it can alleviate NADH accumulation by redox cycling with it, thereby regenerating NAD^+^, which is crucial in cell growth and human health [76,77].

Peruzzo et al. [12] postulated that submicromolar pyocyanin is non-toxic, restores respiration, and enhances ATP production in fibroblasts from patients carrying pathogenic mutations that impair the assembly or stabilization of complex III. Pyocyanin normalized the mitochondrial membrane potential and modestly increased both ROS generation and mitochondrial biogenesis. Based on inspection of those data, we consider PMS to be substantially more efficient in eliciting these beneficial effects, albeit at the cost of producing markedly higher levels of ROS.

Our findings further suggest that PMS might be useful in acute cyanide poisoning. Cyanide blocks complex IV, preventing the reduction of oxygen to water and halting proton pumping out of the matrix [11], leading rapidly to fatal energy failure. The ideal cyanide antidote, as outlined by Hall et al. [78], should act rapidly, neutralize cyanide without impairing oxygen transport or utilization, be safe in prehospital and hospital settings (including smoke inhalation cases), be non-harmful if given to non-poisoned individuals, and be simple to handle and administer. PMS meets many of these criteria, and our data show that it can effectively bypass cyanide-induced complex IV inhibition. Although PMS can effectively reduce oxygen, this action can be counterbalanced by boosting antioxidant defenses—for example, by administering SOD or catalase, or their mimetics such as Mn-based EUK-8 [79], or by using N-acetylcysteine (NAC) [80] alone or with ebselen [81], a synthetic glutathione peroxidase mimic. Current cyanide antidotes (e.g., amyl nitrite + sodium nitrite + sodium thiosulfate (CAK), 4-dimethylaminophenol (4-DMAP)) act within minutes [78], but PMS given intravenously could, in principle, act even faster. Moreover, PMS could be combined with existing antidotes—cyanide binders (e.g., dicobalt edetate), chelators (hydroxocobalamin), or methemoglobin inducers (4-DMAP, CAK)—to provide a multipronged approach to cyanide detoxification. In agreement with previous work [20], PMS up to 10 μM did not confer substantial toxicity in 2 h incubation.

In acute mitochondrial failure scenarios (e.g., cyanide poisoning), PMS administration is envisaged in a low-dose (≤10 μM), short-term, ETC-bypass regimen, rather than the high-dose pro-oxidant regimen (>10 μM) explored for selective cancer cell killing.

## Data Availability

The data supporting the results of this work will be made available from the corresponding author upon reasonable request.

## Supplementary Materials

The following supporting information can be downloaded at: https://www.mdpi.com/article/10.3390/antiox15060749/s1, Figure S1: Unmodified Spectra of bioluminescence corresponding to Figure 2C,D., Figure S2: PMS cytotoxicity., Figure S3: GBM cell viability following long-term incubation with PMS at 1, 2, and 5 μM., Figure S4: Seahorse experiment with incremental PMS additions to T98G cells., Code for bioluminescence graphs in Figure 2 (main manuscript), Video link for video in Figure 3.
