# Supplementary material for: Phenazine Methosulfate Rewires Mitochondrial Redox Circuits to Restore Membrane Potential and ATP Synthesis Under ETC Blockade in Glioblastoma Cells
Source: Antioxidants (Basel). 2026 Jun 13;15(6):749. doi: 10.3390/antiox15060749 (PMC13296177; doi:10.3390/antiox15060749)
Supplement: Supplementary file 1 [file antioxidants-15-00749-s001.zip › antioxidants-4233183-supplementary.pdf]

SUPPLEMENTARY INFORMATION

Phenazine Methosulfate Rewires Mitochondrial Redox Circuits to Restore Membrane Potential and ATP Synthesis under ETC Blockade.

Andrius Kleinauskas 1†, Marianna Canonaco 1†, Tine Therese Henriksen Raabe 2, Elin Ryan 2, Petras Juzenas 2, Beata Grallert 2, Aspasia Valiraki 3, Athanasios Papakyriakou 3, Theodossis A. Theodossiou 1,2*

1 Department of Physics, University of Oslo, PO Box 1048 Blindern, N-0316, Oslo, Norway

2 Department of Radiation Biology, Institute for cancer Research, Oslo University Hospital, Montebello, N-0379 Oslo, Norway

3 Institute of Biosciences & Applications, National Centre for Scientific Research "Demokritos", Ag. Paraskevi, 15341 Athens, Greece.

† The researchers contributed equally to the work

***Corresponding Author to whom correspondence should be sent:** theodoss@uio.no, ththeo@ous-hf.no, t.theodossiou@gmail.com**.**

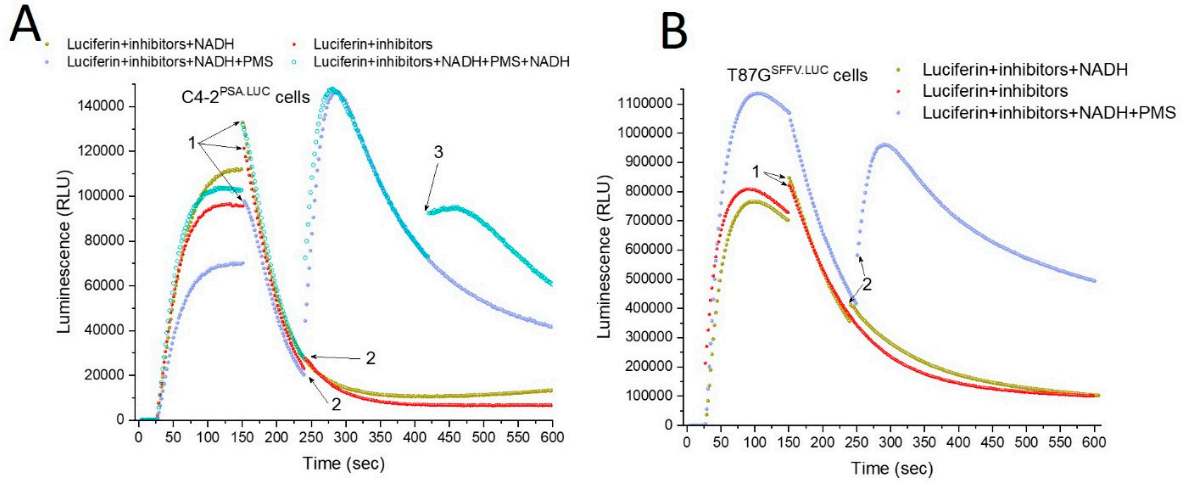

Figure S1. Unmodified Spectra of bioluminescence corresponding to Fig. 2C,D. A. Bioluminescence profile of luciferase-transduced C4-2$^{PSA.LUC}$ cells. Cells were pretreated with 2-DG (50 mM, 1 h) to block glycolytic ATP production. Traces shown are: (a) luciferin (300 µM) plus mitochondrial ETC inhibitors MYXO/ANTIA (complex III) and ROT (complex I) (10 µM each), red spheres; (b) as in (1) with the addition of NADH (20 mM), yellow spheres; (c) as in (a) with the addition of PMS/NADH (10 µM/20 mM), lilac spheres; (d) as in (c) with an extra NADH bolus (200 µM luciferin, 7 µM inhibitors, 8 µM PMS), open cyan circles. D. Bioluminescence profile of luciferase-transduced T87G$^{SFFV.LUC}$ cells. Cells were pretreated with 2-DG (50 mM, 2 h). Traces shown are: (a) luciferin (300 µM) plus mitochondrial ETC inhibitors MYXO/ANTIA (complex III) and ROT (complex I) (10 µM each), red spheres; (b) as in (a) with the addition of NADH (20 mM), dark yellow spheres; (c) as in (a) with the addition of PMS/NADH (10 µM/20 mM), lilac open spheres. The arrows denote the addition of (1) the inhibitors ROT, ANTIA and MYXO, (2) the addition of either NADH or PMS/NADH and (3) extra addition of NADH. The arrows also show the points of either hardware and/or software anomalies leading to spurious jumps in the luminescence intensity, associated with each compound addition.

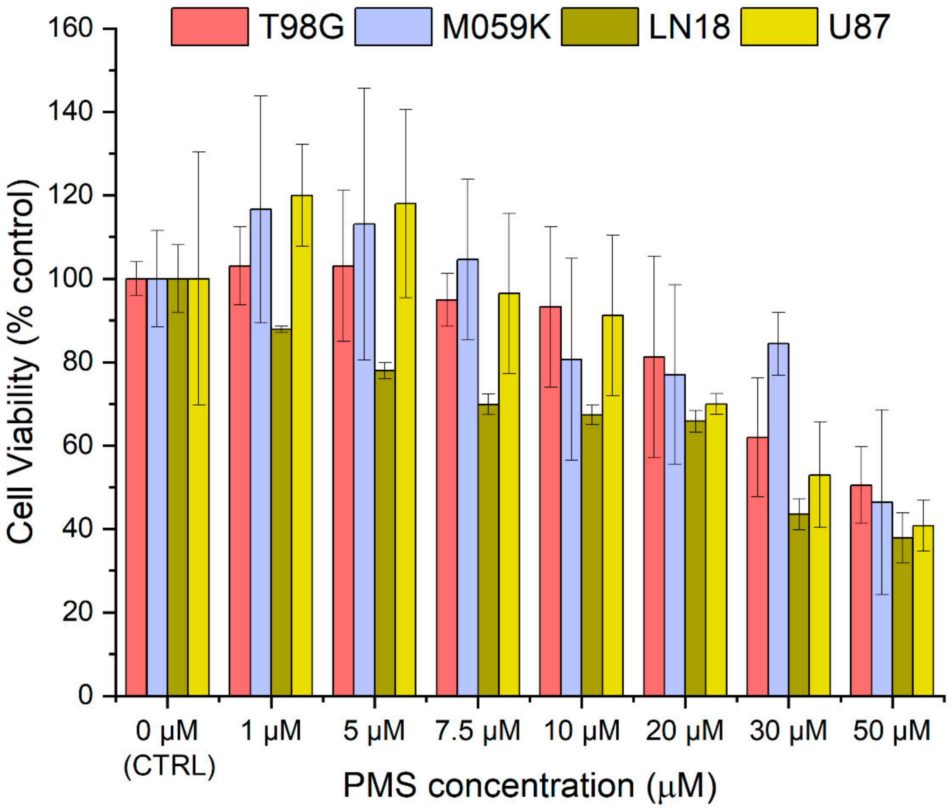

Figure S2. PMS cytotoxicity. Effect of 2 h incubation of GBM cells with increasing concentrations of PMS on cell viability, assessed by a standard MTT assay 24 h after PMS exposure. Error bars represent ±1 standard deviation (SD).

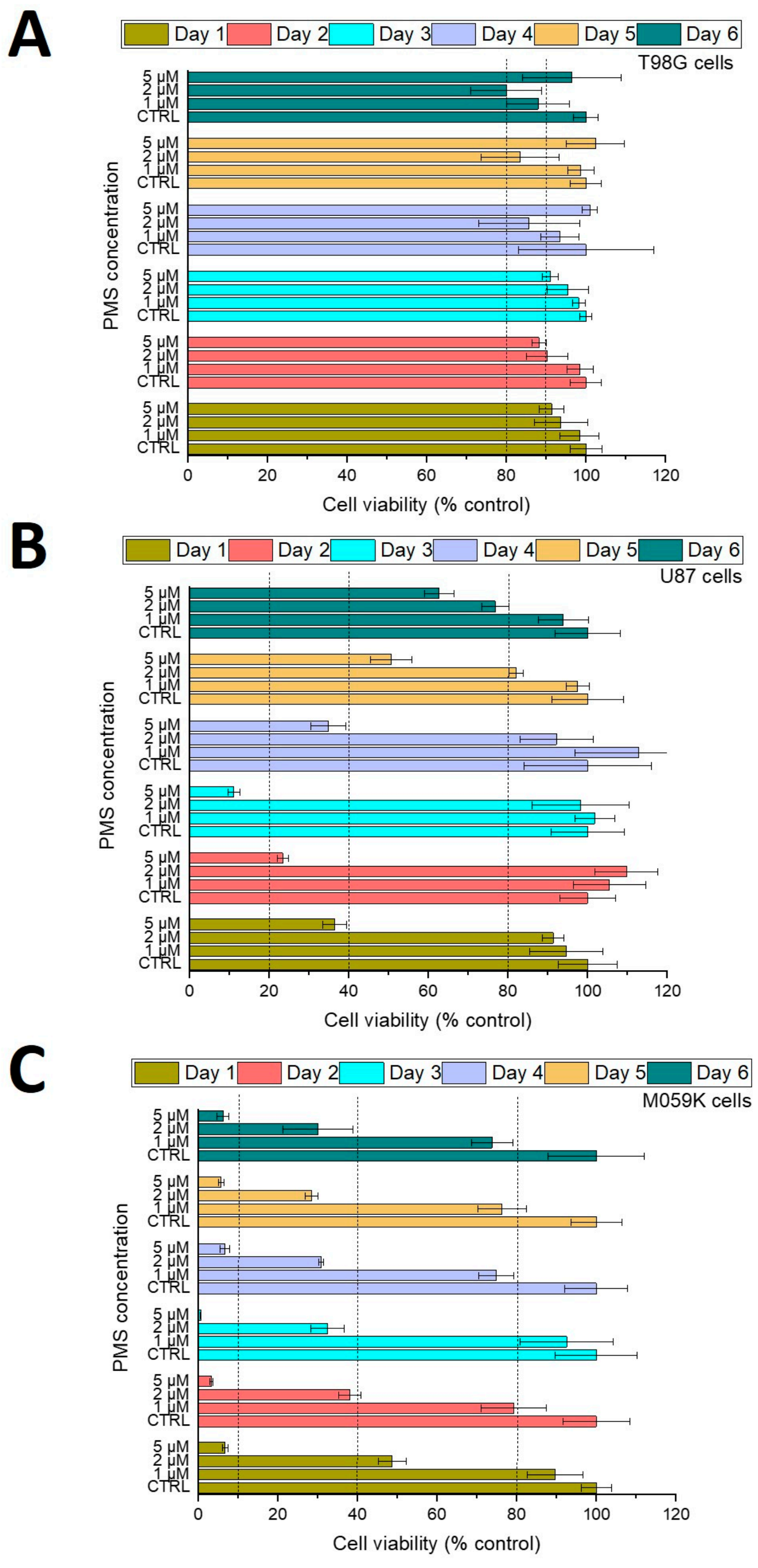

A
Day 1 Day 2 Day 3 Day 4 Day 5 Day 6
T98G cells
5 µM
2 µM
1 µM
CTRL
PMS concentration
Cell viability (% control)
0 20 40 60 80 100 120
B
Day 1 Day 2 Day 3 Day 4 Day 5 Day 6
U87 cells
5 µM
2 µM
1 µM
CTRL
PMS concentration
Cell viability (% control)
0 20 40 60 80 100 120
C
Day 1 Day 2 Day 3 Day 4 Day 5 Day 6
M059K cells
5 µM
2 µM
1 µM
CTRL
PMS concentration
Cell viability (% control)
0 20 40 60 80 100 120

Figure S3. GBM cell viability following long-term incubation with PMS at 1, 2, and 5 µM. Viability was assessed in T98G, U87 and M059K cells at 24, 48, 72, 96, 120, and 144 h, via standard MTT assays, with PMS remaining in the culture medium throughout until each assay timepoint.

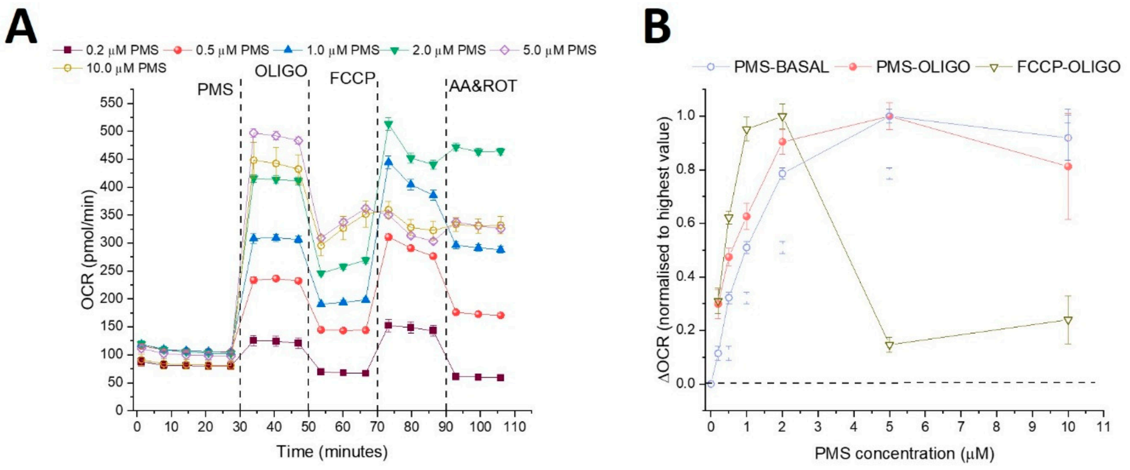

Figure S4. Seahorse experiment with incremental PMS additions to T98G cells. A) OCR traces in basal respiration, after 0.2-10 µM PMS addition, with subsequent addition of 1.5 µM OLIGO, 1.5 µM FCCP and finally 2 µM ANTIA and ROT. B) Normalised data derived from A, illustrating the PMS-BASAL, PMS-OLIGO, and FCCP-OLIGO responses across the various PMS concentrations. This experiment was repeated twice (n=2).

In order to mend the stiching incontinuities in Figs. S1 A,B, we wrote codes for batch processing for C4-2$^{PSA.LUC}$ and T87G$^{SFFV.LUC}$ cells. These codes are shown below:

Code for C4-2$^{PSA.LUC}$ bioluminescence graphs in Fig 2 (main manuscript)

```
"""
RLU time-series processing pipeline

What this script does:
1) Load multiple CSV datasets (time, RLU) and convert time to seconds.
2) Plot an overview with one subplot per dataset.
3) Manually segment each dataset using user-provided (Time_seconds, RLU)
points.
- For each segmentation point, the nearest data
point is located by Euclidean distance in (t, y) space.
- The data is split into segments around those indices.
```

```
4) Normalize each segment by its own max RLU (per-
segment normalization).
5) Plot the original trace and each normalized segment side-by-side.
6) Stitch normalized segments back together into one continuous series:
- Rescale the next segment to match the end of
the previous (to ensure continuity).
- Optionally drop the duplicated junction point.
7) Export selected stitched series to CSV
for OriginLab and save stitched plots as PNGs.

Assumptions:
- Input CSVs have a usable "Time" column (HH:MM:SS or HH:MM:SS.sss)
and an "RLU" column.
- There is header/skip rows, handled by pd.read_csv parameters below.

Outputs:
- Plotly window for quick overview.
-
A series of Matplotlib figures showing original + segments and stitched results.

- CSV files for selected labels in "ExportingData4OriginLab_csv/".
- PNG images of stitched plots in the working directory.

"""
```

```
import pandas as pd
import plotly.subplots as sp
import plotly.graph_objects as go
import numpy as np
import matplotlib.pyplot as plt
import os
```

```
# ----------------------------
# Utility functions
# ----------------------------

# Converting into sec
def time_to_seconds(t):
h, m, s = t.split(":")
return int(h) * 3600 + int(m) * 60 + float(s)
```

```
def closest_index(df, t, y):
"""
Find the index in df that is closest to the point (t, y) using
Euclidean distance in (Time_seconds, RLU) space.
"""
dist = np.sqrt((df["Time_seconds"] - t)**2 + (df["RLU"] - y)**2)
return dist.idxmin()
def segment_and_normalize(df, points):
"""
Split df into segments around the closest indices to the provided (t, y) points
and produce a new list of DataFrames, each with a new column "RLU_norm"
that is normalized by the segment's own max RLU.
"""
# 1) Finding the indeces
indices = [closest_index(df, t, y) for (t, y) in points]
indices = sorted(indices)

# 2) intervals for the segments
segments = []
start = 0
for idx in indices:
seg = df.iloc[start:idx+1].copy()
seg["RLU_norm"] = seg["RLU"] / seg["RLU"].max()
segments.append(seg)
start = idx+1

# 3) Last segment till the end
seg = df.iloc[start:].copy()
seg["RLU_norm"] = seg["RLU"] / seg["RLU"].max()
segments.append(seg)

return segments
def plot_segments(label, df, segments):
"""
Plot one row:
- Column 1: original (Time_seconds vs RLU)
- Columns 2..N: each segment normalized (Time_seconds vs RLU_norm)

Parameters:
label: Dataset label for plot titles.
df: Original DataFrame.
```

```
segments: List of segment DataFrames with "RLU_norm".
"""

n_seg = len(segments)
fig, axs = plt.subplots(n_seg+1, 1, figsize=(3, 2.2*(n_seg+1)))

# Original
axs[0].plot(df["Time_seconds"], df["RLU"], lw=2)
axs[0].set_title(f"{label} – Original")
axs[0].set_ylabel("RLU")

# Segments
for i, seg in enumerate(segments, start=1):
axs[i].plot(seg["Time_seconds"], seg["RLU_norm"], lw=2)
axs[i].set_title(f"Segment {i} (normalized)")
axs[i].set_ylabel("Norm")
axs[-1].set_xlabel("Time (s)")

plt.tight_layout()
plt.show()
import matplotlib.pyplot as plt

def plot_row(label, df, segments):
n_seg = len(segments)
total_cols = n_seg + 1 # original + segments

fig, axs = plt.subplots(1, total_cols, figsize=(4*total_cols, 3))

# If only one column
if total_cols == 1:
axs = [axs]

# --- Column 1: original ---
axs[0].plot(df["Time_seconds"], df["RLU"], lw=2)
axs[0].set_title(f"{label}\nOriginal")
axs[0].set_xlabel("Time (s)")
axs[0].set_ylabel("RLU")

# --- Segments ---
for i, seg in enumerate(segments):
axs[i+1].plot(seg["Time_seconds"], seg["RLU_norm"], lw=2)
axs[i+1].set_title(f"Segment {i+1}")
```

```
axs[i+1].set_xlabel("Time (s)")
axs[i+1].set_ylabel("Norm")

plt.tight_layout()
plt.show()

def stitch_and_rescale(segments, atol=1e-6):


"""
Stitch a list of normalized segments into a single continuous series.

Strategy:
- Start with the first segment as-is.
- For each subsequent segment:
* Compare previous end value (prev_y_end)
with current segment start value (curr_start).
* If they differ (beyond atol), rescale the entire current segment by factor = pre
v_y_end / curr_start
so that the junction is continuous.
* If the first point of the current segment equals the previous end (within atol),
drop it to avoid duplicates.

Parameters:
segments: List of DataFrames with columns ["Time_seconds", "RLU_norm"].
atol: Absolute tolerance for "is close" checks.

Returns:
(stitched_time, stitched_norm) as numpy arrays.
"""

stitched_t = segments[0]["Time_seconds"].values.copy()
stitched_y = segments[0]["RLU_norm"].values.copy()

for i in range(1, len(segments)):
prev_y_end = stitched_y[-1]

curr_seg = segments[i]
curr_y = curr_seg["RLU_norm"].values.copy()
curr_t = curr_seg["Time_seconds"].values.copy()

curr_start = curr_y[0]
```

```
# --- Rescaling if necessary ---
if not np.isclose(prev_y_end, curr_start, atol=atol):
scale = prev_y_end / curr_start
curr_y *= scale

# --- If the junction is the same, delete the first point ---
if np.isclose(prev_y_end, curr_y[0], atol=atol):
curr_y = curr_y[1:]
curr_t = curr_t[1:]

# --- Append ---
stitched_t = np.concatenate([stitched_t, curr_t])
stitched_y = np.concatenate([stitched_y, curr_y])

return stitched_t, stitched_y
```

```
# Data and labels
file_label_pairs = [
("1-Liuci-Mixo-Rot-AA.csv", "Luci+Myxo&Rot&AA"),
("4-Liuci-Mixo-Rot-AA-NADH-PMS-NADH-
NADH.csv", "Luci+Myxo&Rot&AA+NADH+PMS+NADH+NADH"),
("6-Liuci-Mixo-Rot-AA-NADH-
PMS.csv", "Luci+Myxo&Rot&AA+NADH+PMS3rd"),
("8-Liuci-Mixo-Rot-AA-NADH.csv", "Luci+Myxo&Rot&AA+NADH2nd")
]

#Directory to save the data
out_dir = "ExportingData4OriginLab_csv"
os.makedirs(out_dir, exist_ok=True)

# Loading the data
dataframes = {}
# Dataset
for file, label in file_label_pairs:
df = pd.read_csv(file, skiprows=[1], sep=",", header=1, engine='python')
df = df.iloc[:, :2]
df.columns = ["Time", "RLU"]
df["Time_seconds"] = df["Time"].apply(time_to_seconds)
df["RLU"] = pd.to_numeric(df["RLU"], errors='coerce')
dataframes[label] = df
```

```
# --- Setup subplot ---
n = len(dataframes)
cols = 3
rows = (n + cols - 1) // cols
fig = sp.make_subplots(rows=rows, cols=cols, subplot_titles=list(dataframes.k
eys()))

# --- Adding the trace for each condition ---
for idx, (label, df) in enumerate(dataframes.items()):
row = idx // cols + 1
col = idx % cols + 1
fig.add_trace(go.Scatter(x=df["Time_seconds"], y=df["RLU"], mode='lines+mar
kers',
name=f"{label} Corrected", opacity=0.9),
row=row, col=col)
fig.update_xaxes(title_text="Time (s)", row=row, col=col)
fig.update_yaxes(title_text="RLU", row=row, col=col)

# --- Layout finale ---
fig.update_layout(height=300 * rows, width=1300,
title_text="RLU Trends per Condition", showlegend=False)
fig.show()


#Saving the data
for label, df in dataframes.items():
out_df = df[["Time_seconds", "RLU"]].copy()
out_df.columns = ["Time_seconds", "RLU"]
fname = f"RLU_{label}.csv"
out_df.to_csv(os.path.join(out_dir, fname), index=False)
```

```
#I got manually all the points where to cut the graphs
segment_points = {

"Luci+Myxo&Rot&AA": [
(148.61, 112130)
],
```

```
"Luci+Myxo&Rot&AA+NADH2nd": [
(150.54, 95693),
(293.32, 23093)
],
"Luci+Myxo&Rot&AA+NADH+PMS+NADH+NADH": [
(148.61, 102784),
(293.32, 27509),
(418.81, 73162),
(600.23, 60476)
],
"Luci+Myxo&Rot&AA+NADH+PMS3rd": [
(150.54, 70215),
(239.32, 20376)
]
}

all_segments = {}

for label, df in dataframes.items():

if label in segment_points:
points = segment_points[label]
segments = segment_and_normalize(df, points)
else:
print(f"⚠ No segmentation for {label} → I use the whole plot")
seg = df.copy()
seg["RLU_norm"] = seg["RLU"] / seg["RLU"].max()
segments = [seg]

all_segments[label] = (df, segments)
plot_row(label, df, segments)
```

```
#Data I need to export And save for Origin Lab
export_labels = {
"Luci+Myxo&Rot&AA",
"Luci+Myxo&Rot&AA+NADH+PMS+NADH+NADH",
"Luci+Myxo&Rot&AA+NADH+PMS3rd",
"Luci+Myxo&Rot&AA+NADH2nd"
}
```

```
#Directory to save the data
os.makedirs(out_dir, exist_ok=True)

# ---- LOOP ON ALL THE DATASET ----
for label, (df, segments) in all_segments.items():

t_st, y_st = stitch_and_rescale(segments)

plt.figure(figsize=(9,4))
plt.plot(t_st, y_st, lw=2)
plt.title(f"{label} – stitched & rescaled")
plt.xlabel("Time (s)")
plt.ylabel("Normalized RLU (-)")
plt.grid(alpha=0.3)
plt.tight_layout()
# ---- SAVING CSV ----
if label in export_labels:

out_df = pd.DataFrame({
"Time_seconds": t_st,
"RLU_norm": y_st
})

fname = f"Contiuos_{label}_C42_28Aug2025.csv".replace("&","_").replace("+",
"_").replace(" ","_")
out_df.to_csv(os.path.join("ExportingData4OriginLab_csv", fname), index=Fal
se)

plt.savefig(f"{label}.png", dpi=300, format='png')
plt.show()
```

Code for $T87G^{SFFV.LUC}$ bioluminescence graphs in Fig yD (main manuscript)

```
"""
RLU time-series processing pipeline

What this script does:
1) Load multiple CSV datasets (time, RLU) and convert time to seconds.
2) Plot an overview with one subplot per dataset.
3) Manually segment each dataset using user-provided (Time_seconds, RLU)
points.
```

```
- For each segmentation point, the nearest data
point is located by Euclidean distance in (t, y) space.
- The data is split into segments around those indices.
4) Normalize each segment by its own max RLU (per-
segment normalization).
5) Plot the original trace and each normalized segment side-by-side.
6) Stitch normalized segments back together into one continuous series:
- Rescale the next segment to match the end of
the previous (to ensure continuity).
- Optionally drop the duplicated junction point.
7) Export selected stitched series to CSV
for OriginLab and save stitched plots as PNGs.

Assumptions:
- Input CSVs have a usable "Time" column (HH:MM:SS or HH:MM:SS.sss)
and an "RLU" column.
- There is header/skip rows, handled by pd.read_csv parameters below.

Outputs:
- Plotly window for quick overview.
-
A series of Matplotlib figures showing original + segments and stitched results.

- CSV files for selected labels in "ExportingData4OriginLab_csv/".
- PNG images of stitched plots in the working directory.

"""
```

```
import pandas as pd
import plotly.subplots as sp
import plotly.graph_objects as go
import numpy as np
import matplotlib.pyplot as plt
import os
```

```
# ---------------------------
# Utility functions
# ---------------------------

# Converting into sec
def time_to_seconds(t):
```

```
h, m, s = t.split(":")
return int(h) * 3600 + int(m) * 60 + float(s)
def closest_index(df, t, y):
"""
Find the index in df that is closest to the point (t, y) using
Euclidean distance in (Time_seconds, RLU) space.
"""
dist = np.sqrt((df["Time_seconds"] - t)**2 + (df["RLU"] - y)**2)
return dist.idxmin()
def segment_and_normalize(df, points):
"""
Split df into segments around the closest indices to the provided (t, y) points
and produce a new list of DataFrames, each with a new column "RLU_norm"
that is normalized by the segment's own max RLU.
"""
# 1) Finding the indeces
indices = [closest_index(df, t, y) for (t, y) in points]
indices = sorted(indices)

# 2) intervals for the segments
segments = []
start = 0
for idx in indices:
seg = df.iloc[start:idx+1].copy()
seg["RLU_norm"] = seg["RLU"] / seg["RLU"].max()
segments.append(seg)
start = idx+1

# 3) Last segment till the end
seg = df.iloc[start:].copy()
seg["RLU_norm"] = seg["RLU"] / seg["RLU"].max()
segments.append(seg)

return segments
def plot_segments(label, df, segments):
"""
Plot one row:
- Column 1: original (Time_seconds vs RLU)
- Columns 2..N: each segment normalized (Time_seconds vs RLU_norm)

Parameters:
```

```
label: Dataset label for plot titles.
df: Original DataFrame.
segments: List of segment DataFrames with "RLU_norm".
"""

n_seg = len(segments)
fig, axs = plt.subplots(n_seg+1, 1, figsize=(3, 2.2*(n_seg+1)))

# Original
axs[0].plot(df["Time_seconds"], df["RLU"], lw=2)
axs[0].set_title(f"{label} – Original")
axs[0].set_ylabel("RLU")

# Segments
for i, seg in enumerate(segments, start=1):
axs[i].plot(seg["Time_seconds"], seg["RLU_norm"], lw=2)
axs[i].set_title(f"Segment {i} (normalized)")
axs[i].set_ylabel("Norm")
axs[-1].set_xlabel("Time (s)")

plt.tight_layout()
plt.show()
import matplotlib.pyplot as plt

def plot_row(label, df, segments):
n_seg = len(segments)
total_cols = n_seg + 1 # original + segments

fig, axs = plt.subplots(1, total_cols, figsize=(4*total_cols, 3))

# If only one column
if total_cols == 1:
axs = [axs]

# --- Column 1: original ---
axs[0].plot(df["Time_seconds"], df["RLU"], lw=2)
axs[0].set_title(f"{label}\nOriginal")
axs[0].set_xlabel("Time (s)")
axs[0].set_ylabel("RLU")

# --- Segments ---
for i, seg in enumerate(segments):
```

```
axs[i+1].plot(seg["Time_seconds"], seg["RLU_norm"], lw=2)
axs[i+1].set_title(f"Segment {i+1}")
axs[i+1].set_xlabel("Time (s)")
axs[i+1].set_ylabel("Norm")

plt.tight_layout()
plt.show()

def stitch_and_rescale(segments, atol=1e-6):


"""
Stitch a list of normalized segments into a single continuous series.

Strategy:
- Start with the first segment as-is.
- For each subsequent segment:
* Compare previous end value (prev_y_end)
with current segment start value (curr_start).
* If they differ (beyond atol), rescale the entire current segment by factor = pre
v_y_end / curr_start
so that the junction is continuous.
* If the first point of the current segment equals the previous end (within atol),
drop it to avoid duplicates.

Parameters:
segments: List of DataFrames with columns ["Time_seconds", "RLU_norm"].
atol: Absolute tolerance for "is close" checks.

Returns:
(stitched_time, stitched_norm) as numpy arrays.
"""

stitched_t = segments[0]["Time_seconds"].values.copy()
stitched_y = segments[0]["RLU_norm"].values.copy()

for i in range(1, len(segments)):
prev_y_end = stitched_y[-1]

curr_seg = segments[i]
curr_y = curr_seg["RLU_norm"].values.copy()
curr_t = curr_seg["Time_seconds"].values.copy()
```

```
curr_start = curr_y[0]

# --- Rescaling if necessary ---
if not np.isclose(prev_y_end, curr_start, atol=atol):
scale = prev_y_end / curr_start
curr_y *= scale

# --- If the junction is the same, delete the first point ---
if np.isclose(prev_y_end, curr_y[0], atol=atol):
curr_y = curr_y[1:]
curr_t = curr_t[1:]

# --- Append ---
stitched_t = np.concatenate([stitched_t, curr_t])
stitched_y = np.concatenate([stitched_y, curr_y])

return stitched_t, stitched_y
```

```
# Lista dei file e relative etichette
file_label_pairs = [
("100000_50000uM 2DG 2h 3min incub time_600uM Luc at 25sec_80uL
AA_ROT_MYX at 2.30 min 60uL 100mM NADH at 4 min
B.csv", "Luci+Myxo&Rot&AA+NADH"),
("100000_50000uM 2DG 2h 3min incub time_600uM Luc at 25sec_80uL
AA_ROT_MYX at 2.30 min 60uL 100mM NADH-PMS 10uM at 4
min.csv", "Luci+Myxo&Rot&AA+NADH+PMS"),
("100000_50000uM 2DG 2h 3min incub time_600uM Luc at 25sec_80uL
AA_ROT_MYX at 2.30 min B.csv", "Luci+Myxo&Rot&AA2nd")
]
#Directory to save the data
out_dir = "ExportingData4OriginLab_csv"
os.makedirs(out_dir, exist_ok=True)
# Loading the data
dataframes = {}
# Dataset
for file, label in file_label_pairs:
df = pd.read_csv(file, skiprows=[1], sep=",", header=1, engine='python')
df = df.iloc[:, :2]
df.columns = ["Time", "RLU"]
df["Time_seconds"] = df["Time"].apply(time_to_seconds)
```

```
df["RLU"] = pd.to_numeric(df["RLU"], errors='coerce')
dataframes[label] = df
# --- Setup subplot ---
n = len(dataframes)
cols = 3
rows = (n + cols - 1) // cols
fig = sp.make_subplots(rows=rows, cols=cols, subplot_titles=list(dataframes.k
eys()))

# --- Aggiunta trace per ogni condizione ---
for idx, (label, df) in enumerate(dataframes.items()):
row = idx // cols + 1
col = idx % cols + 1
fig.add_trace(go.Scatter(x=df["Time_seconds"], y=df["RLU"], mode='lines+mar
kers',
name=f"{label} Corrected", opacity=0.9),
row=row, col=col)
fig.update_xaxes(title_text="Time (s)", row=row, col=col)
fig.update_yaxes(title_text="RLU", row=row, col=col)

# --- Layout finale ---
fig.update_layout(height=300 * rows, width=1300,
title_text="RLU Trends per Condition", showlegend=False)
fig.show()

#Saving the data
for label, df in dataframes.items():
out_df = df[["Time_seconds", "RLU"]].copy()
out_df.columns = ["Time_seconds", "RLU"]
fname = f"RLU_{label}.csv"
out_df.to_csv(os.path.join(out_dir, fname), index=False)
```

```
#I got manually all the points where to cut the graphs
segment_points = {

"Luci+Myxo&Rot&AA+NADH": [
(148.61, 702062),
(293.32, 356814)
],
```

```
"Luci+Myxo&Rot&AA+NADH+PMS": [
(248.97, 417405)
],
"Luci+Myxo&Rot&AA2nd": [
(148.61, 728723)
]
}

all_segments = {}

for label, df in dataframes.items():

if label in segment_points:
points = segment_points[label]
segments = segment_and_normalize(df, points)
else:
print(f"⚠ Nessuna segmentazione per {label} → uso curva intera")
seg = df.copy()
seg["RLU_norm"] = seg["RLU"] / seg["RLU"].max()
segments = [seg]

all_segments[label] = (df, segments)
plot_row(label, df, segments)
```

```
#Exporting the graph for OriginLab
export_labels = {
"Luci+Myxo&Rot&AA+NADH",
"Luci+Myxo&Rot&AA+NADH+PMS",
"Luci+Myxo&Rot&AA2nd"
}
#Directory where I save the data for OriginLab
os.makedirs(out_dir, exist_ok=True)
# ---- LOOP SU TUTTI I DATASET ----
for label, (df, segments) in all_segments.items():

t_st, y_st = stitch_and_rescale(segments)

plt.figure(figsize=(9,4))
plt.plot(t_st, y_st, lw=2)
plt.title(f"{label} – stitched & rescaled")
plt.xlabel("Time (s)")
```

```
plt.ylabel("Normalized RLU (-)")
plt.grid(alpha=0.3)
plt.tight_layout()
plt.savefig(f"{label}.png", dpi=300, format='png')
# ---- SALVATAGGIO CSV SOLO PER I LABEL SELEZIONATI ----
if label in export_labels:

out_df = pd.DataFrame({
"Time_seconds": t_st,
"RLU_norm": y_st
})

fname = f"Continuous{label}_T98G864_25Aug2025.csv".replace("&","_").replace("+","_").replace(" ","_")
out_df.to_csv(os.path.join("ExportingData4OriginLab_csv", fname), index=False)

plt.show()
```

The cyt c reduction video that was analysed in Fig. 3 can be found in the following link:

https://uio-my.sharepoint.com/:f:/g/personal/marican_uio_no/IgAYuuL1oAkXTZVZDW_9g02AAU3WQvX67PffZ-jNaak1jwY?e=OyfogO
